# The effect of shingles vaccination at different stages of the dementia disease course

**DOI:** 10.1016/j.cell.2025.11.007

**Published:** 2025-12-02

**Authors:** Min Xie, Markus Eyting, Christian Bommer, Haroon Ahmed, Pascal Geldsetzer

**Affiliations:** 1 Heidelberg Institute of Global Health, Heidelberg University Hospital, 69120 Heidelberg, Germany; 2 Division of Primary Care and Population Health, Department of Medicine, Stanford University, Stanford, CA 94305, USA; 3 Faculty of Law and Economics, Johannes Gutenberg University Mainz, 55122 Mainz, Germany; 4 Leibniz Institute for Financial Research SAFE, 60323 Frankfurt am Main, Germany; 5 Division of Population Medicine, School of Medicine, Cardiff University, Cardiff CF14 4YS, UK; 6 The Phil and Penny Knight Initiative for Brain Resilience at the Wu Tsai Neurosciences Institute, Stanford University, Stanford, CA 94305, USA; 7 Stanford Center for Digital Health, Stanford University, Stanford, CA 94305, USA; 8 Biohub, San Francisco, CA 94158, USA; 9 Lead contact

## Abstract

Using natural experiments, we have previously reported that live-attenuated herpes zoster (HZ) vaccination appears to have prevented or delayed dementia diagnoses in both Wales and Australia. Here, we find that HZ vaccination also reduces mild cognitive impairment diagnoses and, among patients living with dementia, deaths due to dementia. Exploratory analyses suggest that the effects are not driven by a specific dementia type. Our approach takes advantage of the fact that individuals who had their eightieth birthday just after the start date of the HZ vaccination program in Wales were eligible for the vaccine for 1 year, whereas those who had their eightieth birthday just before were ineligible and remained ineligible for life. The key strength of our natural experiments is that these comparison groups should be similar in all characteristics except for a minute difference in age. Our findings suggest that live-attenuated HZ vaccination prevents or delays mild cognitive impairment and dementia and slows the disease course among those already living with dementia.

## In brief

A natural experiment found that herpes zoster vaccination reduced the occurrence of mild cognitive impairment and deaths due to dementia, indicating that the vaccine may slow the progression of dementia throughout its clinical disease course.

## Graphical Abstract

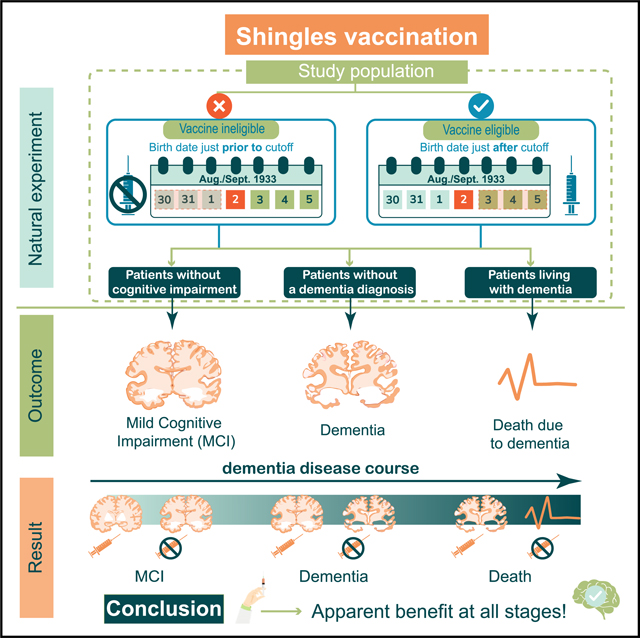

## INTRODUCTION

Given the key role of neuroinflammation in the development and progression of dementia,^1^ it is conceivable that neurotropic viruses could be a factor that causes or accelerates the dementia disease process. Neurotropic herpesviruses have thus far received the greatest research attention in this regard,^2–4^ partly because they remain latent for life in the nervous system after primary infection, are more likely to reactivate with increasing age, and can cause encephalitis.^5^ Recently, several findings have further spurred interest in neurotropic herpesviruses, including the observation that they can seed β-amyloid in mice^6^ and increase tau phosphorylation in human brain organoids^7^ and that the Epstein-Barr virus appears to be a causative factor in the development of multiple sclerosis.^8^

Clinical and subclinical reactivations of the neurotropic herpesvirus (the varicella zoster virus) that causes chickenpox and shingles may constitute a chronic immune stressor that drives inflammatory pathways in both the peripheral and central nervous system, interfering with neuroimmune homeostasis in older age.^9^ The varicella zoster virus has also recently been linked to amyloid deposition and aggregation of tau proteins,^10^ as well as cerebrovascular disease that resembles the patterns commonly seen in Alzheimer’s disease, such as small- to large-vessel disease, ischemia, infarction, and hemorrhage.^11–16^ Reducing clinical and subclinical reactivations of the virus through herpes zoster (HZ) vaccination might thus have a beneficial impact on the development or progression of dementia, as well as neuroimmune health and cognitive reserve in older age more broadly. Moreover, as has been detailed recently elsewhere,^17^ it is possible that HZ vaccination, and potentially vaccinations in older age more generally, act on the dementia disease process through a pathogen-independent immune mechanism. Such an effect might counteract immunosenescence^17,18^ and would add to the growing body of evidence suggesting that vaccines frequently have broader health benefits beyond their intended target.^19–22^ Of importance with respect to this present study, these beneficial off-target effects have often been found to be far stronger among female than male individuals,^20^ as well as for live-attenuated rather than other types of vaccines.^19–21,23^

In a recent study,^24^ we were able to take advantage of a unique quasi-randomization in Wales to provide evidence of the effect of HZ vaccination on new dementia diagnoses that is more likely to be causal than the previously existing associational evidence. This opportunity arose because the UK National Health Service rolled out the live-attenuated HZ vaccine (Zostavax, Merck) using strict date-of-birth-based eligibility rules.^25^ These rules resulted in an increase in the probability of ever receiving the HZ vaccine of almost 50 percentage points between individuals who differed in age by merely a week across the date-of-birth-based eligibility threshold for the vaccination program. We thus had the opportunity to compare dementia incidence between eligible and ineligible groups of individuals who were not expected to differ in their characteristics other than a difference in age of merely a few weeks and a large difference in ever having received the HZ vaccine. We found that HZ vaccination averted an estimated one in five new dementia diagnoses over a 7-year follow-up period. Crucially, unlike the previously existing associational evidence,^26–35^ this study is not subject to the fundamental concern in associational studies that those who opt to be vaccinated differ from those who do not in a variety of characteristics that are difficult to measure.^36^ Most recently, taking advantage of a similar date-of-birth-based rollout of HZ vaccination in Australia, we have shown that this protective effect for new diagnoses of dementia from live-attenuated HZ vaccination also appears to exist in the Australian population.^37^

To guide further research in this area and, ultimately, inform appropriate clinical care, it is critical to understand at which stage of the disease course of dementia HZ vaccination has its benefit. Our previous analyses in Wales and Australia have left this question unanswered. The aims of this study, therefore, were 2-fold: to determine the effect of HZ vaccination on (1) new diagnoses of mild cognitive impairment (MCI) among individuals without any record of cognitive impairment and (2) deaths due to dementia among individuals living with dementia. In the absence of more widespread testing for β-amyloid and tau pathology during the study period (2013–2022), these two aims represent the two opposite ends of the disease course of dementia (considering the limitations in ascertaining different disease stages in electronic health record data). Here, we report a large beneficial effect from live-attenuated HZ vaccination for both aims, suggesting that the vaccine appears to act across the entire clinical disease course of dementia.

## RESULTS

### Sample characteristics

We used the Secure Anonymised Information Linkage (SAIL) databank.^38^ Our dataset, which consisted of electronic health record data from approximately 80% of Wales’s primary care practices,^39^ was linked by SAIL to hospital-based care data as well as death certificate data (Table 1). Our dataset consisted of 304,940 individuals born between September 1, 1925, and September 1, 1942, who were alive and residing in Wales as of September 1, 2013. Of these individuals, 282,557 did not have a record of any cognitive impairment prior to September 1, 2013, and were thus included in our study cohort for analyzing the effect of HZ vaccination on MCI. Our study cohort for analyzing the effect of HZ vaccination on deaths due to dementia consisted of the 14,350 individuals in our dataset who had received a diagnosis of dementia prior to September 1, 2013. The sample characteristics of each of these two cohorts are shown in Table 2.

### A 1-week difference in age led to a large difference in HZ vaccination uptake

**Figure 1.**
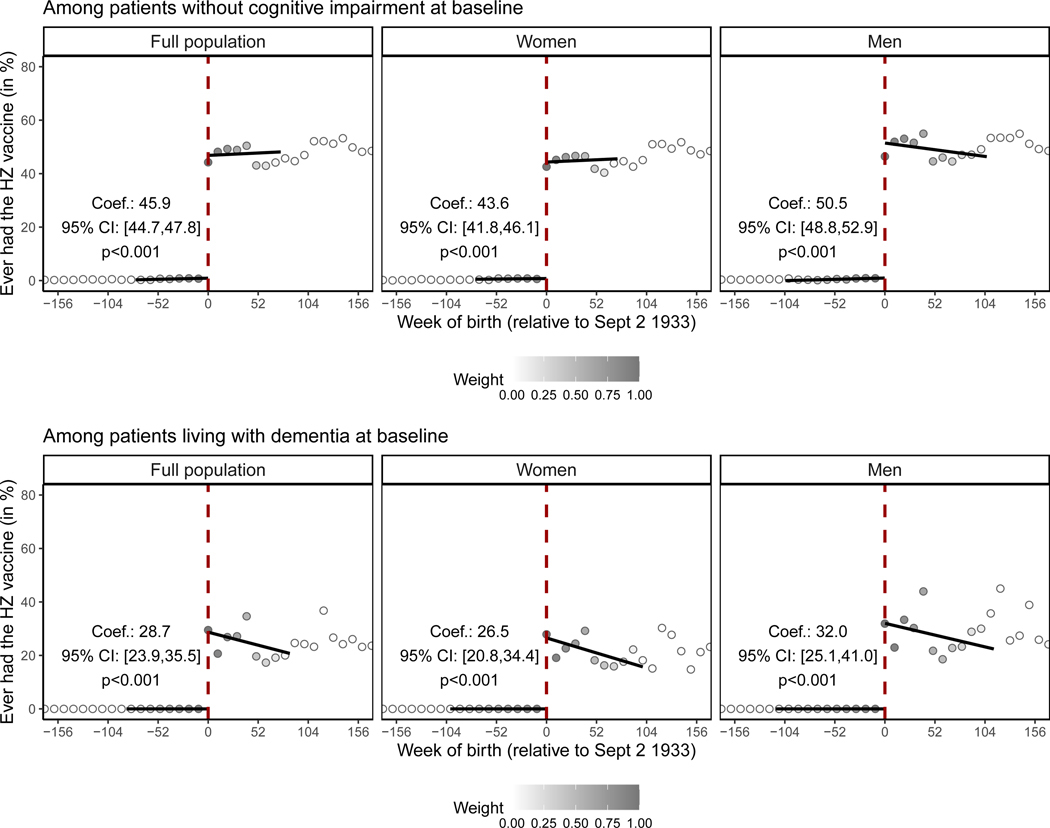
The abrupt change in the probability of receiving the HZ vaccination at the September 2, 1933, date-of-birth eligibility threshold “Baseline” refers to the start date of the HZ vaccination program (i.e., September 1, 2013). Linear regression lines were drawn in the mean squared error-optimal bandwidth only. Gray dots show the mean value for each 10-week increment in week of birth. The gray shading of the dots is in proportion to the weight that observations from this 10-week increment received in the analysis. Abbreviations are as follows: HZ, herpes zoster; Coef., coefficient; CI, confidence interval; Sept, September. See also Figure S10.

We analyzed our data using a regression discontinuity design, which is a well-established causal effect estimation technique that is guided by the expectation that individuals born just on either side of the date-of-birth eligibility threshold for HZ vaccination likely are similar to each other except for their probability of receiving the HZ vaccine.^41–43^ In both of our study cohorts, a 1-week difference in age across the September 2, 1933, date-of-birth eligibility threshold resulted in a large difference in the probability of ever receiving the HZ vaccination (Figures 1 and S10). Specifically, among individuals without any record of cognitive impairment prior to the start of the HZ vaccination program, being born 1 week after September 2, 1933, and thus being eligible for HZ vaccination, led to an abrupt increase in the probability of ever receiving the HZ vaccination from 0.0% to 45.9% (*p* < 0.001). The corresponding abrupt increase among patients living with dementia on the start date of the HZ vaccination program was from 0.0% to 28.7% (*p* < 0.001). Thus, as expected, in both of our study cohorts, the eligibility rules of the HZ vaccination program created comparison groups born just on either side of the September 2, 1933, date-of-birth threshold who (after flexibly controlling for age) were likely similar to each other except for a large difference in the probability of receiving the HZ vaccination.

Prior to the start date of the HZ vaccination program, there were no significant differences at the September 2, 1933, date-of-birth threshold in the uptake of preventive health services, the prevalence of any of the ten most common causes of disability-adjusted life years (DALYs) and mortality among adults aged 70+ years in Wales (except ischemic heart disease among those living with dementia at program start),^44^ or in the occurrence of HZ, diagnoses of MCI, and deaths due to dementia (Figure S1).

### A protective effect of HZ vaccination on new diagnoses of MCI

To determine the effect of being eligible for HZ vaccination on our outcomes, our analytical strategy focused on comparing individuals who were not eligible for the HZ vaccine because they turned 80 years of age immediately before the program’s start date with those who were eligible because they turned 80 years of age immediately after the start date. Following standard regression discontinuity methods,^41,42^ we estimated the effect of actually receiving the vaccine (rather than merely being eligible) using a two-stage least-squares regression. This approach divides the size of the sudden change in the outcome at the date-of-birth eligibility cutoff by the size of the sudden change in vaccine uptake at that same cutoff. As a result, the fact that not everyone who was eligible actually received the vaccine does not introduce bias into our analysis.

**Figure 2.**
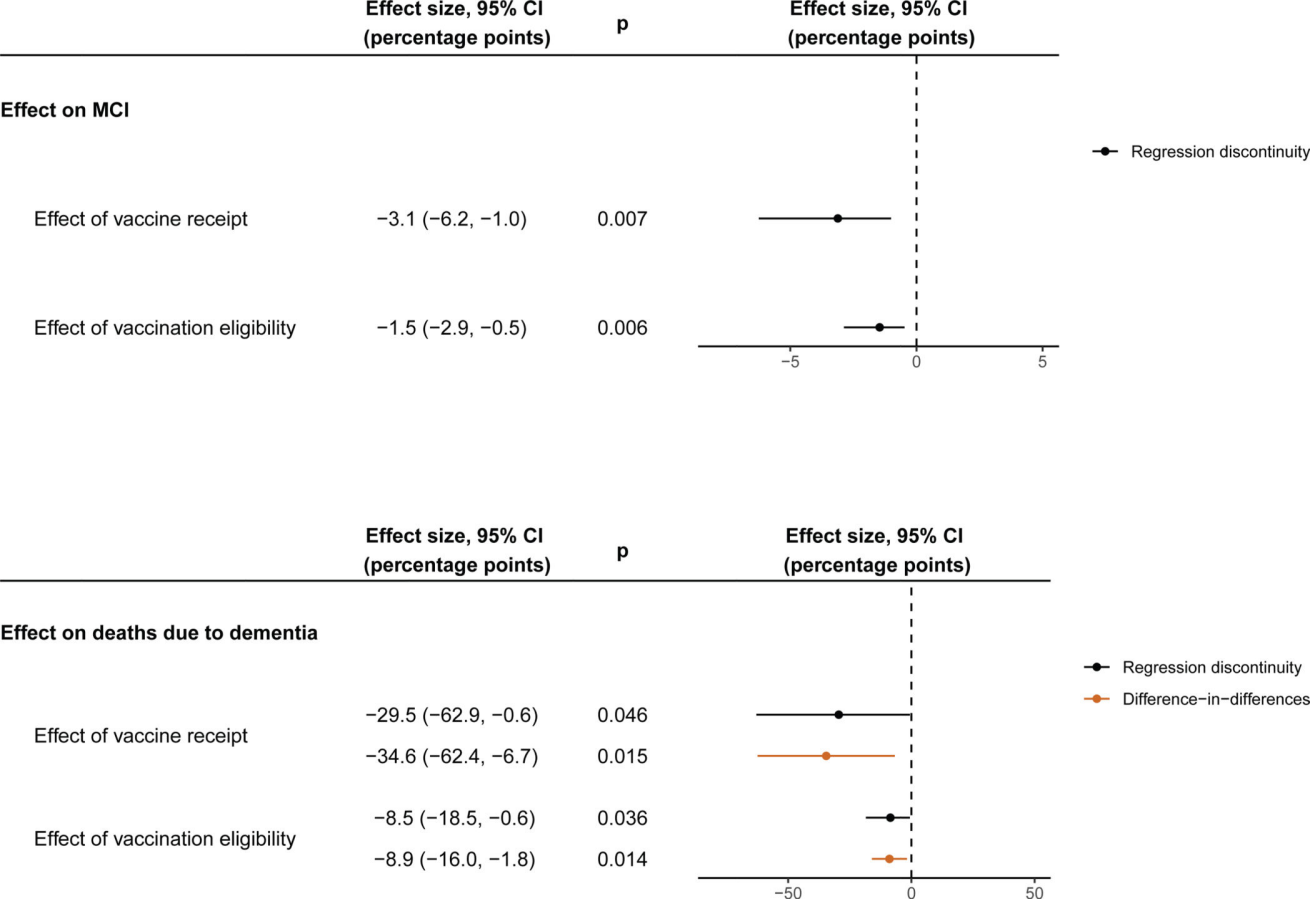
The effect of HZ vaccination on new diagnoses of MCI and deaths due to dementia Dots show the point estimate and horizontal bars the 95% CI. New diagnoses of MCI were analyzed among a study cohort of patients who did not have any record of cognitive impairment prior to the start date of the HZ vaccination program. Deaths due to dementia were analyzed among a study cohort of patients who had received a diagnosis of dementia prior to the start date of the HZ vaccination program. Abbreviations are as follows: MCI, mild cognitive impairment; CI, confidence interval. See also Figures S2–S4.

Among our study cohort of individuals without any record of cognitive impairment prior to the start date of the HZ vaccination program, 20,712 (7.3%) were newly diagnosed with MCI during our 9-year follow-up period. Being born immediately after versus immediately before September 2, 1933, and thus being eligible for HZ vaccination, decreased the occurrence of new diagnoses of MCI over 9 years by 1.5 (95% confidence interval [CI]: 0.5–2.9, *p* = 0.006) percentage points (Figure 2). Scaled to the proportion of individuals who took up HZ vaccination if they were eligible using our regression discontinuity approach, the effect of actually receiving the HZ vaccination was a 3.1 (95% CI: 1.0–6.2, *p* = 0.007) percentage point reduction in new diagnoses of MCI over 9 years. The effect across different follow-up periods is shown in Figure S2. Both the effect of being eligible for HZ vaccination and the effect of actually receiving the HZ vaccination were robust across different choices of bandwidth, grace period, and functional form (using local quadratic instead of local linear regression), when requiring that a new MCI diagnosis not be followed by a new dementia diagnosis within 0–3 months or within 0–6 months, when adjusting for the staggered rollout of the program, when adjusting for indicators of health service utilization, and when restricting the analysis cohort to frequent primary care visitors (Figure S3).

### A protective effect of HZ vaccination on deaths due to dementia

Among our study cohort of individuals with a diagnosis of dementia received prior to the start date of the HZ vaccination program, 7,049 (49.1%) died due to dementia (i.e., had dementia recorded as the underlying cause of death in their death certificate) over the 9-year follow-up period. Being eligible for HZ vaccination (i.e., being born shortly after versus shortly before September 2, 1933) decreased the occurrence of deaths due to dementia over 9 years by 8.5 (95% CI: 0.6–18.5, *p* = 0.036) percentage points (Figure 2). The effect of actually receiving the HZ vaccination was a 29.5 (95% CI: 0.6–62.9, *p* = 0.046) percentage point reduction in deaths due to dementia over 9 years. Our difference-in-differences analysis (detailed in the STAR Methods and Figure S11) yielded similar results as our regression discontinuity analysis (Figure 2). The effect across different follow-up periods is shown in Figure S2. As for MCI as outcome, the point estimates for the effect of being eligible for HZ vaccination and the effect of actually receiving the HZ vaccination were robust across different choices of bandwidth, grace period, and functional form (using local quadratic instead of local linear regression) and when adjusting for the staggered rollout of the program (Figure S3). In addition, the effect remained significant when adjusting for the baseline imbalance in ischemic heart diseases diagnoses using a fully non-parametric regression discontinuity method that can flexibly control for such baseline imbalances (Figure S3).^45^

### Evidence against the existence of confounding

Given the focus of our regression discontinuity approach on changes in the outcome variable at the date-of-birth eligibility threshold, a confounding variable would only bias our analysis if it changed abruptly at the September 2, 1933, date-of-birth threshold. Such bias could arise if another intervention used the identical date-of-birth eligibility threshold (i.e., September 2, 1933) as the HZ vaccination program. As detailed in the STAR Methods, we tested for this possibility in four ways.

First, the September 2 date-of-birth eligibility threshold only had a significant effect on our outcomes in the birth year (1933) that was used by the HZ vaccination program but not in any of the 3 birth years prior to and after 1933 (Figure S4). This finding reduces the probability that an annual intervention existed that also used September 2 as a date-of-birth eligibility criterion.

Second, at the time of the start date of the HZ vaccination program, we did not observe systematic differences across the September 2, 1933, date-of-birth threshold in past preventive health services uptake nor the prevalence of the ten leading causes of DALYs and mortality among adults aged 70+ years in Wales (Figure S1). Third, there were no significant differences in the incidence of new MCI diagnoses (in our study cohort for analyzing the effect on MCI) and deaths due to dementia (in our study cohort for analyzing the effect on deaths due to dementia) across the September 2, 1933, date-of-birth threshold in the 9 years before the start of the HZ vaccination program (MCI: 0.0 [95% CI: − 0.5 to 0.6, *p* = 0.953] percentage points; deaths due to dementia: − 5.2 [− 17.7 to 6.7, *p* = 0.375] percentage points). Together, these tests reduce the likelihood that a competing intervention (i.e., another intervention that used the identical date-of-birth eligibility threshold as the HZ vaccination program) existed that was implemented prior to the HZ vaccination program.

**Figure 3.**
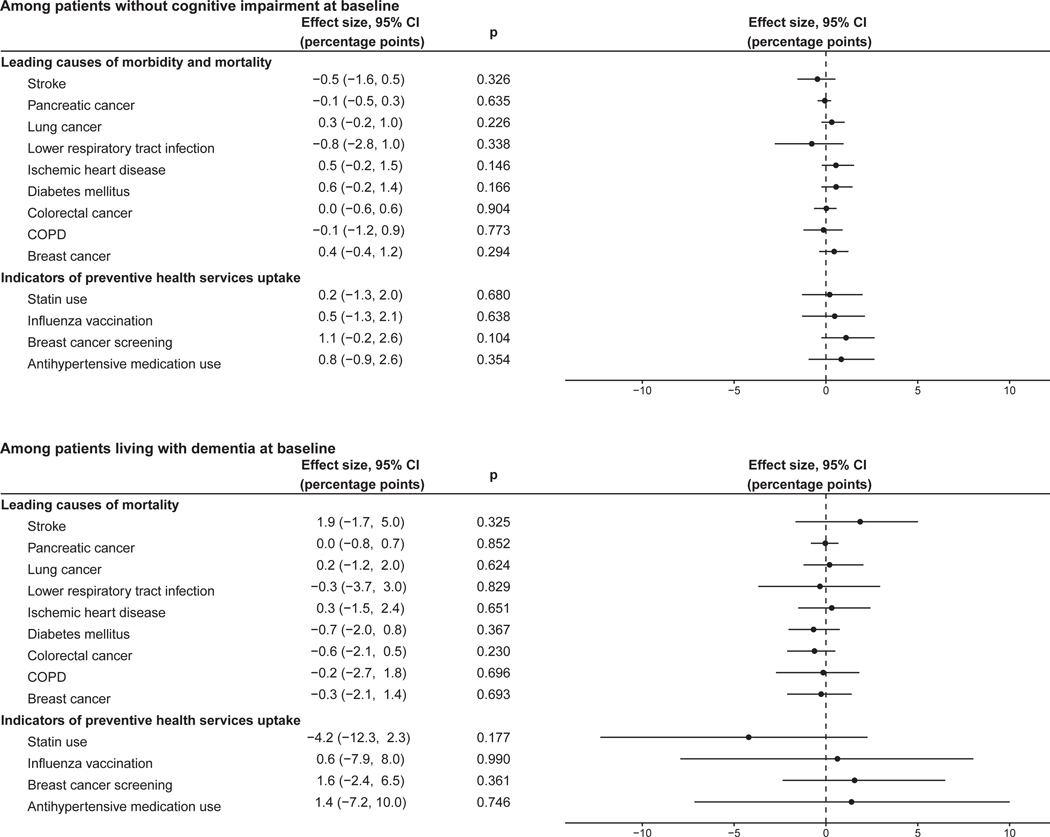
No significant effect of being eligible for HZ vaccination on the leading causes of morbidity and mortality (other than dementia) nor on indicators of preventive health services uptake Baseline refers to the start date (September 1, 2013) of the HZ vaccination program. Dots show the point estimate and horizontal bars the 95% CI. Among patients without a record of cognitive impairment prior to the start date of the HZ vaccination program, the leading causes of morbidity and mortality were the ten (other than dementia) leading causes of DALYs and mortality among adults aged 70+ years in Wales, as estimated by the Global Burden of Disease Project.^44^ Among patients with a diagnosis of dementia made prior to the start date of the HZ vaccination program, the leading causes of mortality were the ten (other than dementia) leading causes of mortality among adults aged 70+ years in Wales, as estimated by the Global Burden of Disease Project.^44^ Among patients without a record of cognitive impairment prior to the start date of the HZ vaccination program, a new diagnosis of a given condition was defined as the diagnosis being recorded for the first time in our electronic health record data or as an underlying or contributing cause on the death certificate. Among patients with a diagnosis of dementia made prior to the start date of the HZ vaccination program, a death was considered to be caused by a given condition if it was recorded as the underlying cause of death on the death certificate. Influenza vaccination was defined as receipt of influenza vaccination at any time in the 12 months after the start date of the HZ vaccination program. Statin and antihypertensive medication use was defined as any prescription of these medications during the 12 months after the start date of the HZ vaccination program. Breast cancer screening and diagnoses were analyzed among women only. Breast cancer screening was defined as a record of referral to, attendance at, or a report from “breast cancer screening” or mammography at any time after the start date of the HZ vaccination program. The Read and ICD (International Classification of Diseases) codes used to define each variable shown in this figure are provided in Data S1. Abbreviations are as follows: COPD, chronic obstructive pulmonary disease; CI, confidence interval.

Fourth, other than for dementia, we did not observe any significant effects of the September 2, 1933, date-of-birth eligibility threshold on diagnoses of each of the ten most common causes of DALYs and mortality (in our study cohort for analyzing the effect on MCI) and deaths due to the ten leading causes of mortality (in our study cohort for analyzing the effect on deaths due to dementia) among adults aged 70+ years in Wales over our 9-year follow-up period (Figure 3). Neither did we observe any effects on indicators of preventive health services uptake during our follow-up period (Figure 3). These tests provide evidence against the existence of an intervention that used the identical date-of-birth eligibility threshold as the HZ vaccination program and was not specifically designed to only affect our primary outcomes.

### The protective effects were larger among women than men

**Figure 4.**
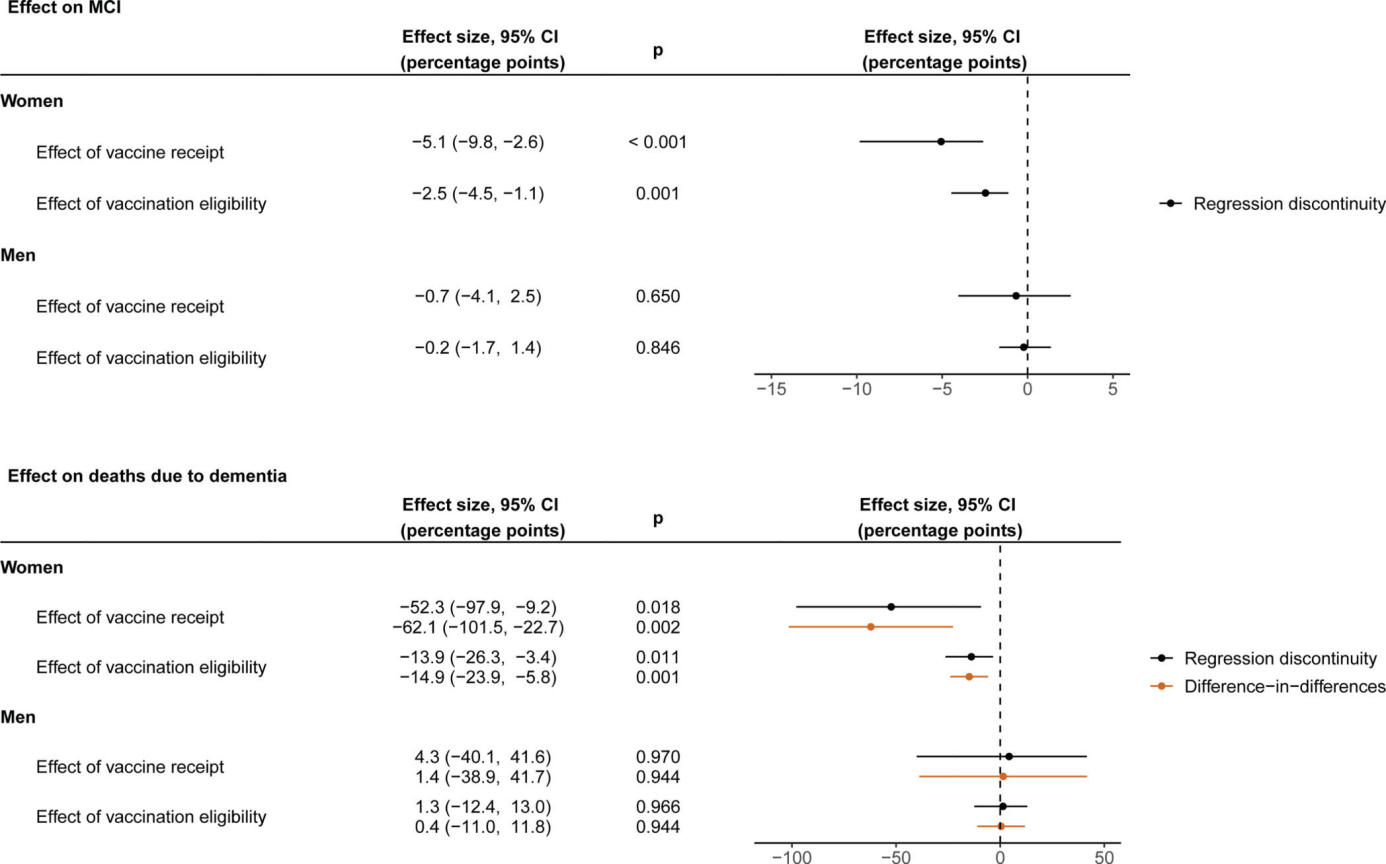
The effect of HZ vaccination on new diagnoses of MCI and deaths due to dementia separately by gender Dots show the point estimate and horizontal bars the 95% CI. New diagnoses of MCI were analyzed among a study cohort of patients who did not have any record of cognitive impairment prior to the start date of the HZ vaccination program. Deaths due to dementia were analyzed among a study cohort of patients who had received a diagnosis of dementia prior to the start date of the HZ vaccination program. Abbreviations are as follows: MCI, mild cognitive impairment; CI, confidence interval. See also Figures S5–S7.

The effect of HZ vaccination—both on reducing new diagnoses of MCI and deaths due to dementia—was larger among women than men (Figure 4). Among women, the effects of being eligible for, and actually receiving, HZ vaccination on new diagnoses of MCI were a reduction of 2.5 (95% CI: 1.1–4.5, *p* = 0.001) and 5.1 (95% CI: 2.6–9.8, *p* < 0.001) percentage points, respectively. The corresponding estimates for the effects on deaths due to dementia among women living with dementia at baseline were a decrease of 13.9 (95% CI: 3.4–26.3, *p* = 0.011) and 52.3 (95% CI: 9.2–97.9, *p* = 0.018) percentage points. For both MCI and deaths due to dementia, the estimates among men were statistically indistinguishable from zero. Formal interaction tests by gender showed that the interaction was significant for both new MCI diagnoses (*p* = 0.029) and deaths due to dementia (*p* = 0.039) (Table S1). The significant effects among women were robust across different choices of bandwidth, grace period, and functional form (using local quadratic instead of local linear regression), as well as when requiring that a new MCI diagnosis not be followed by a new dementia diagnosis within 0–3 months or within 0–6 months, adjusting for the staggered rollout of the program, adjusting for indicators of health service utilization, and restricting the analysis cohort to frequent primary care visitors (Figure S5).

To examine whether the benefits of HZ vaccination in reducing deaths due to dementia also apply to those who already have more advanced dementia, we additionally conducted a set of subgroup analyses that divided those living with dementia at the time of the start date of the HZ vaccination program into those with more versus less severe dementia. To do so, we used several indicators—as measured prior to September 1, 2013—that we hypothesized likely reflect dementia severity: (1) risk of all-cause mortality, as predicted from each condition that is part of the Charlson comorbidity index^46^; (2) the total number of days spent as an inpatient in hospital (as part of admissions for which dementia was recorded as a primary or secondary reason); (3) the number of hospital admissions (for which dementia was recorded as a primary or secondary reason); (4) the number of times that a diagnosis of dementia was recorded in the electronic health record from primary, secondary, or tertiary care; and (5) risk of death due to dementia, as predicted from the variables defined in (2) and (4), as well as each condition that is part of the Charlson comorbidity index.^46^ Although not reaching statistical significance, directionally we found tentative evidence that the protective effect, both on an absolute and relative scale, of HZ vaccination on deaths due to dementia was greater for those with more advanced dementia (Figures S6 and S7).

### Exploratory analyses by type of dementia

We conducted a set of exploratory analyses to provide suggestive evidence of which type of dementia is most strongly affected by HZ vaccination. To do so, we focused on women because our heterogeneous treatment effect analyses presented in the preceding section found that effects were largely driven by this group. Given that different dementia types vary in their incidence, we compared relative rather than absolute effects. This required a focus on point estimates because there is no established method to estimate CIs and *p* values for relative effects in a regression discontinuity approach.^42^ We relied on hospital-based diagnoses as opposed to primary care diagnoses because these are more likely to be based on a thorough assessment that also includes brain imaging.^47^ To maintain adequate sample sizes, we limited our comparison to diagnoses of the three most common types of dementia in our data, which were Alzheimer’s disease, vascular dementia, and mixed dementia. Our analyses examined the effect of HZ vaccination receipt and eligibility on dementia diagnoses by type for both women without a dementia diagnosis at baseline (i.e., the same analysis cohort as used in Eyting et al.^24^) as well as those without cognitive impairment at baseline (i.e., the same analysis cohort as used for our MCI outcome analysis). We also examined only those dementia diagnoses that were made subsequent to an MCI diagnosis during the follow-up period. As shown in Table S2, the relative reductions in dementia incidence from HZ vaccination receipt and eligibility were generally largest for diagnoses of mixed dementia as opposed to diagnoses of Alzheimer’s disease and diagnoses of vascular dementia.

### The protective effect for dementia deaths resulted in a reduction in all-cause mortality

**Figure 5.**
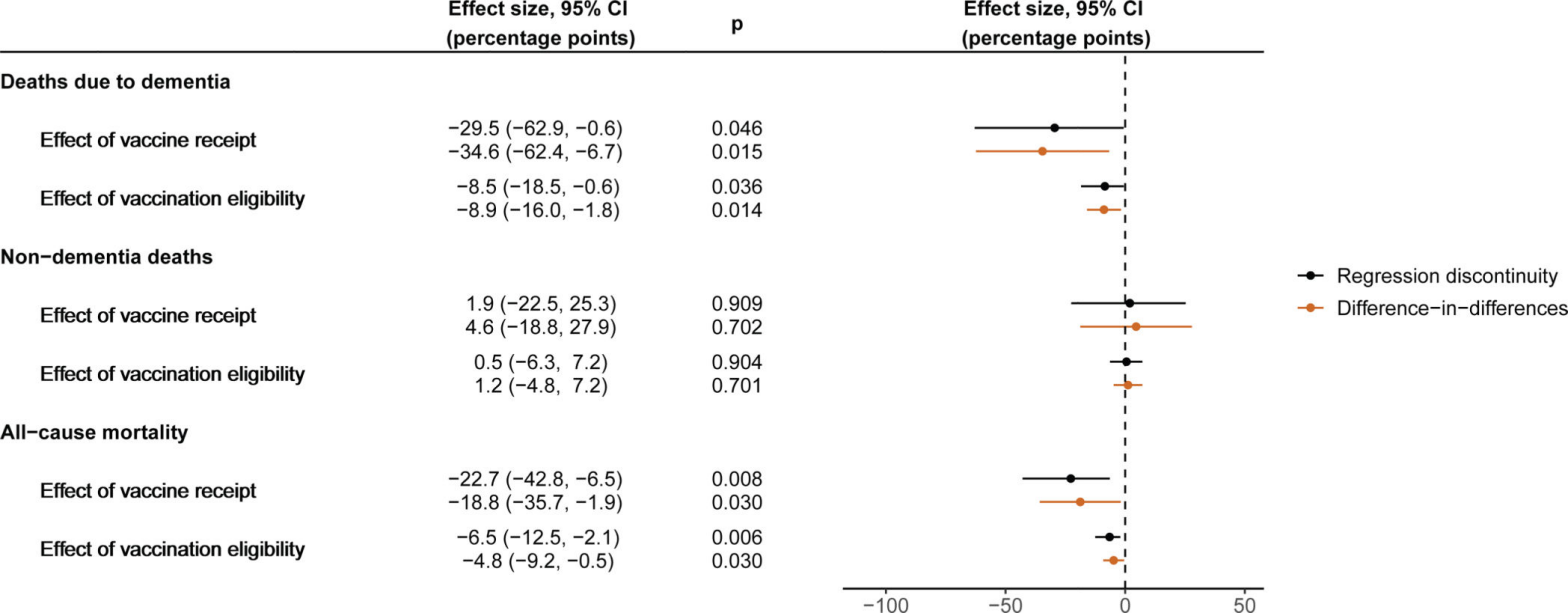
The effect of HZ vaccination on deaths due to dementia, non-dementia deaths, and all-cause mortality Dots show the point estimate and horizontal bars the 95% CI. Deaths due to dementia were analyzed among a study cohort of patients who had received a diagnosis of dementia prior to the start date of the HZ vaccination program. Non-dementia deaths were defined as deaths for which dementia was recorded as neither the underlying nor a contributing cause of death in the death certificate. Abbreviation is as follows: CI, confidence interval. See also Figures S8 and S9.

Among patients living with dementia at baseline, being eligible for HZ vaccination (based on being born immediately after versus immediately before September 2, 1933) decreased all-cause mortality over our 9-year follow-up period by 6.5 (95% CI: 2.1–12.5, *p* = 0.006) percentage points (Figure 5). Adjusted for the proportion who took up HZ vaccination if they were eligible using our regression discontinuity approach, the effect of actually receiving the HZ vaccination was a 22.7 (95% CI: 6.5–42.8, *p* = 0.008) percentage point reduction in all-cause mortality over 9 years. There was no significant effect of HZ vaccination eligibility or receipt on non-dementia deaths. As for deaths due to dementia, the effects of HZ vaccination eligibility and receipt on all-cause mortality were larger among women and statistically indistinguishable from zero among men (Figure S8). All results were similar when using a difference-in-differences instead of a regression discontinuity approach. Both among the whole sample and among women only, the effect on all-cause mortality was robust across different choices of bandwidth and grace period, as well as when using local quadratic instead of local linear regression—adjusting for the staggered rollout of the program—and when adding a dichotomous covariate that indicated whether an individual had been diagnosed with ischemic heart disease prior to the start date of the HZ vaccination program (Figure S9).

## DISCUSSION

We found that live-attenuated HZ vaccination reduced both new diagnoses of MCI among those without any record of cognitive impairment and deaths due to dementia among patients living with dementia. The HZ vaccine thus appears to have a beneficial effect at both ends of the clinical disease course of dementia. Although our point estimates indicate large effect sizes, the exact magnitude of the effect was difficult to ascertain in this analysis, given the wide CIs around our estimates. The lower statistical power compared with standard associational analyses was a result of our focus on individuals born in close proximity to the September 2, 1933, date-of-birth threshold.

Our findings suggest that HZ vaccination could be an effective intervention to prevent or delay MCI and dementia, as well as to reduce disease progression among those living with dementia. Such a beneficial effect also appears to exist among those who already have more advanced dementia, based on our finding that the reductions in deaths due to dementia from HZ vaccination tended to be larger among those with more versus less severe dementia. Using the same quasi-randomization approach as in this study, our group has previously shown that HZ vaccination reduced new diagnoses of dementia in both Wales as well as Australia.^24,37^ This effect could have been observed as a result of HZ vaccination decreasing the transition from cognitively unimpaired to MCI, from MCI to dementia, or (because of the delay in diagnosing dementia in the health system^48–52^) from undiagnosed to diagnosed dementia. This present study suggests that the HZ vaccine acts along the entire clinical disease course of dementia and thus that our previously reported effects were due to a decrease in the transition along each of these dementia disease stages.

Given that it is a readily available, relatively inexpensive,^53–55^ one-off, and safe intervention, the finding that HZ vaccination has a beneficial effect on the dementia disease process would be of great significance to population health, clinical medicine, and dementia research. Thus, confirming our previously reported findings from Wales and Australia is critical. In our view, it is key that such confirmatory studies utilize natural experiments because more standard associational analyses may lead to false confirmations as a result of confounding, such as the healthy vaccinee bias (i.e., the common observation that healthier, more health-motivated individuals opt to be vaccinated^36^). Our quasi-randomized approach is far less vulnerable to these biases because health status, health-related motivation, and other dementia-related characteristics are unlikely to differ between individuals born just before versus just after a specific date-of-birth eligibility threshold. Therefore, in addition to its principal finding that the HZ vaccine appears to act along the entire clinical disease course of dementia, an important contribution of this present study is that it confirms our previously reported findings in two different study populations (those without any record of cognitive impairment and those living with dementia) and using two different dementia-related outcomes (MCI and deaths due to dementia). Deaths with dementia as their primary cause among individuals living with dementia is a particularly opportune outcome in this regard because it is directly related to dementia but less reliant on a timely diagnosis of dementia in the health system, given that dementia is likely to be readily apparent by the time that it is the primary cause of death. Similarly, our secondary outcome of all-cause mortality among individuals living with dementia at baseline is entirely independent of the health system’s process for diagnosing dementia. These mortality outcomes are thus largely measured differently than new diagnoses of dementia. In our view, being able to confirm the benefits of HZ vaccination for dementia using a set of outcomes that are all related to dementia but measured differently, strengthens the evidence that HZ vaccination has an effect on the dementia disease process itself rather than only its measurement (e.g., a diagnostic pathway).

We found that, among individuals living with dementia at baseline, HZ vaccination did not only lead to a decrease in deaths due to dementia but also a reduction in overall mortality. Specifically, we observed a decrease, which was larger among women than men, in both deaths due to dementia and all-cause mortality, but no effect on deaths for which dementia was not mentioned as the underlying or a contributing cause on the death certificate. Our findings thus imply that HZ vaccination among individuals living with dementia increased remaining life expectancy. This reduction in deaths due to dementia is unlikely to be a result of averted shingles episodes, given that shingles has a low mortality rate.^56^ Instead, this study suggests that the HZ vaccine may slow dementia disease progression. Nonetheless, identifying the exact mechanism for this effect is, in our view, an important area of future research.

For each of our outcomes, we found that the protective effect of HZ vaccination was larger among women than men. We observed the same gender effect heterogeneity in our previous study in Wales for new diagnoses of dementia.^57^ However, although none of our results were statistically significant among men, we cannot exclude the possibility of substantial beneficial effects among men as well, given the width of our CIs. A strong gender effect heterogeneity, with beneficial effects usually being more pronounced among females, has frequently been observed for off-target effects of vaccines.^20^ Our observed effect heterogeneity between women and men may thus reflect immunological sex differences. These immunological sex differences may be pathogen independent but could also be specific to the interaction of the immune system with the varicella zoster virus.^58^ The occurrence of shingles has, for instance, been reported to be more common among women in several studies.^59,60^ Additionally, it is equally possible that our observed gender effect heterogeneity reflects differences in the pathophysiology of some types of dementia between women and men: an area that has received increasing research interest in recent years.^61–63^ We did not observe a significant gender effect heterogeneity in our analysis of primary care data from Australia.^37^ We believe that this is likely due to limited statistical power, stemming from the fact that, compared with our data from Wales, our Australian data contained substantially fewer patients, the abrupt increase in the probability of receiving the HZ vaccination at the date-of-birth eligibility threshold was smaller, and the incidence of new dementia diagnoses in the data was lower.

The key strength of this study is its quasi-randomized design. Prior to our analysis in Wales, all epidemiological studies on the relationship between vaccines and dementia had simply compared vaccine recipients with non-recipients while attempting to control for the myriad of characteristics that differ between those who opt to be vaccinated versus those who do not.^26–35^ Electronic health record data do not have detailed information on health behaviors—such as physical activity and diet^64,65^— that are known to be linked to other health behaviors (including vaccination) as well as dementia. These studies are thus vulnerable to confounding.^66^ Our approach is fundamentally different in that we compare individuals who were ineligible or eligible for HZ vaccination because they were born just before or just after the date-of-birth eligibility threshold (September 2, 1933) for HZ vaccination. On average, individuals in Wales born in one week versus merely a week later would not be expected to differ in their health characteristics and behaviors. In the case of the September 2, 1933, threshold, however, there was a large difference in the probability of ever receiving the HZ vaccine between these groups, who differed in age by merely a week. The eligibility rules of the HZ vaccination program in Wales thus created two comparison groups—just on either side of the September 2, 1933, threshold—who were (after flexibly controlling for age) likely similar to each other except for their probability of receiving the intervention of interest (HZ vaccination). As a result, the findings from our analysis are more likely to reflect a causal relationship than are those from the associational epidemiological evidence (Figures S10 and S11).^26–35,67,68^

The critical advantage of our quasi-randomization approach is that a confounding variable can only bias our analysis if it changes abruptly at the September 2, 1933, date-of-birth threshold.^41,42^ Such an abrupt change in a confounding variable at the September 2, 1933, date-of-birth threshold might exist if there were another intervention that used the identical date-of-birth threshold as its eligibility criterion as did the HZ vaccination program. We conducted a series of tests to investigate whether the existence of such an intervention is likely. Specifically, individuals born just on either side of the September 2, 1933, date-of-birth threshold were well-balanced at baseline in their past preventive health services uptake, prevalence of common causes of morbidity and mortality, and past incidence of MCI and deaths due to dementia. Similarly, there was no effect of the September 2, 1933, date-of-birth threshold on common health outcomes (other than dementia) during our 9-year follow-up period, nor on preventive health services uptake indicators (other than HZ vaccination). Lastly, the September 2 date-of-birth threshold only had an effect on MCI and deaths due to dementia in the birth year (1933) that was used by the HZ vaccination program as its eligibility criterion. Thus, none of our tests suggested the existence of another intervention that also used the date of birth of September 2, 1933, as its eligibility criterion. It is in our view also unlikely that HZ vaccination led to increased uptake of other preventive health services (e.g., the uptake of other vaccines at the same visit) because we did not observe any effect of HZ vaccination eligibility on available indicators of preventive health services among older adults in our data.

We believe that our repeated findings from natural experiment studies of a beneficial effect of HZ vaccination on the dementia disease process call for further investments in this area of research, not only to confirm the effects but also to elucidate the mechanisms. Although only suggestive, our exploratory analyses showing larger relative reductions for mixed dementia than for Alzheimer’s disease and vascular dementia point to a mechanism that is at play in both Alzheimer’s disease and vascular dementia—and potentially other dementia types as well. Several such potential mechanisms have been outlined recently,^9^ and these suggest that HZ vaccination may protect neuroimmune health in older age more broadly as well as counteracting immunosenescence. There is also evidence that reactivations of the varicella zoster virus can lead to long-lasting cognitive impairment throughvasculopathy,^69,70^ amyloid deposition and aggregation of tau proteins,^10^ and neuroinflammation,^13–16^ as well as a pattern of cerebrovascular disease that is similar to that seen in Alzheimer’s disease, such as small- to large-vessel disease, ischemia, infarction, and hemorrhage.^11–16^ In addition, reactivations of the varicella zoster virus may lead to reactivations of the herpes simplex virus in the brain.^71,72^ This finding, in turn, would link HZ vaccination to the more extensive literature on the herpes simplex virus as a causative factor in the development of dementia.^3,73^ A recent analysis of electronic health record data from the United States found that indicators of a higher burden of reactivations of the varicella zoster virus were associated with an increased risk of dementia.^68^ Specifically, the authors reported a consistent pattern of a higher burden of varicella zoster virus reactivations (as assessed by comparing dementia risk in those with multiple versus single shingles episodes) being associated with a higher dementia risk, whereas a lower burden (as assessed by comparing dementia risk in those who received an HZ vaccine versus those who received a pneumococcal vaccine, two doses versus one dose of the recombinant subunit HZ vaccine, and the live-attenuated versus the recombinant subunit HZ vaccine) was associated with a lower dementia risk. The analysis also found that the protective association of HZ vaccination with dementia risk was largest among women and older patients—groups who are at a higher risk of shingles.^59,60,74^ Importantly, however, although findings were consistent across analyses, each analysis is subject to the risk of residual confounding and thus correlational in nature only. It may also, therefore, be the case that a pathogen-independent immunomodulatory mechanism is involved, as possibly suggested by our repeated finding of a strong effect heterogeneity by gender.^20^ Some of these potential immune mechanisms have recently been described elsewhere.^17,18^

### Limitations of the study

This study has several important limitations. First, our findings pertain to those age groups born near to the September 2, 1933, date-of-birth threshold (primarily those aged 79–80 years). It is possible that the protective effects observed in this study do not exist, or are of a different magnitude, in younger age groups, given that the neuropathology and pathophysiology of dementia varies strongly by age.^75–79^ In addition, the immune system appears to change in a multitude of characteristics during the last decades of life,^80^ which could influence any role that the varicella zoster virus or a virus-independent immunomodulatory effect induced by the live-attenuated HZ vaccine may have in the dementia disease process. Another reason that effects of the HZ vaccine on dementia-related outcomes may differ by age is that the efficacy of the live-attenuated HZ vaccine for preventing HZ is lower in older than in younger age groups.^58,81^ Second, given its implementation using local linear regression, regression discontinuity is only able to reliably estimate absolute as opposed to relative effects.^41,82^ Third, we were limited to information contained in electronic health record and death certificate data to define our study cohorts and outcomes. It is likely that there is considerable underascertainment in our data for cognitive impairment (to define the cognitively unimpaired study cohort for our first aim), dementia (to define the study cohort of individuals living with dementia for our second aim), MCI, and deaths due to dementia. Crucially, however, the degree of underascertainment (as well as any delay in making these diagnoses or in changes in data quality over time) is unlikely to differ between individuals born just before versus just after September 2, 1933. As such, other than potential underestimation of the true benefits of HZ vaccination on an absolute scale, underascertainment of these variables was unlikely to bias our analysis. Fourth, we were also limited to the information available in electronic health record data to define the two stages of the disease course of dementia analyzed in this study. We, for instance, had no information on β-amyloid or tau pathology or results from detailed neuropsychological assessments. Fifth, the COVID-19 pandemic started within our follow-up period and may have delayed new diagnoses of MCI as well as changed mortality rates among individuals living with dementia. However, as for our third limitation, the pandemic affected individuals born just on either side of the September 2, 1933, date-of-birth threshold equally. Sixth, some diagnoses of MCI in our data may have mistakenly been made for individuals who already had mild-to-moderate dementia. We believe that this is unlikely to be a major limitation of our analysis, given that our effect estimates remained significant when requiring that a new diagnosis of MCI not be followed by a new diagnosis of dementia within 3 months or within 6 months. Seventh, we observed an imbalance in past ischemic heart disease diagnoses across the September 2, 1933, threshold among the study cohort for our second aim. We, however, show in Tables S1 and S2 that our results were robust to any adjustment for this imbalance. In our view, this imbalance was likely a chance finding, given that it was the only difference with a *p* value less than 0.05 in 36 (18 among each of our two study cohorts) baseline balance tests that we conducted. Lastly, our results pertain to the live-attenuated HZ vaccine (Zostavax, Merck) only, because the newer recombinant HZ vaccine (Shingrix, GSK) was introduced into the UK’s National Health Service after our follow-up period ended.^83^

## Conclusions

In conclusion, this study suggests that HZ vaccination slows or prevents disease progression across the entire disease course (as far as it can be feasibly ascertained from electronic health record data) of dementia. By taking advantage of the fact that the UK’s National Health Service assigned individuals who differed in their age by just a few weeks to being eligible or ineligible for HZ vaccination based on their date of birth, we were able to generate evidence that is more likely to be causal than those from more standard epidemiological analyses. Our finding that HZ vaccination had a beneficial effect on two different dementia-related outcomes in two different patient samples—and at two opposing ends of the disease course of dementia—thus provides promising evidence that HZ vaccination may prevent or slow the dementia disease process in a substantial proportion of individuals.

### RESOURCE AVAILABILITY

#### Lead contact

Requests for further information and resources should be directed to, and will be fulfilled by, the lead contact, Pascal Geldsetzer (pgeldsetzer@stanford.edu).

#### Materials availability

This study did not generate new unique reagents.

#### Data and code availability

- The data that support the findings of this study are available from the SAIL databank.^38^ Researchers must request access to the data directly from SAIL. The authors have no permission to share the data.
- All statistical analysis code (in R) has been deposited in a publicly accessible OSF repository at https://osf.io/nwz8h/?view_only=231179cb8d7045f3be22b0979634d9c3. All Read and ICD codes to define variables are available in Data S1.
- Any additional information required to reanalyze the data reported in this paper is available from the lead contact upon request.

## STAR★METHODS

### EXPERIMENTAL MODEL AND STUDY PARTICIPANT DETAILS

#### Human participants

##### Allocation to exposure groups based on the herpes zoster vaccine rollout in Wales

Starting on September 1 2013, the National Health Service ([NHS], the United Kingdom’s single-payer single-provider healthcare system^84^) in Wales made the live-attenuated HZ vaccine (Zostavax, Merck) available to a catch-up cohort of individuals using a staggered rollout system based on specific date-of-birth eligibility thresholds.^25^ Individuals who did not yet have their 80^th^ birthday on the start date of the program (i.e., born on or after September 2 1933) were eligible for one year. By contrast, those who had their 80^th^ birthday prior to the program start date (i.e., born before September 2 1933) never became eligible.

Specifically, individuals were categorized into three cohorts based on their date of birth: i) a routine cohort consisting of individuals born on or after September 2 1942 who became eligible when they were 70 years old as of September 1 of a given year of the HZ vaccination program; ii) a catch-up cohort consisting of individuals born between September 2 1933 and September 1 1942 who became eligible when they were 79 years (or 78 years, depending on the year of the program) as of September 1 of a given year of the HZ vaccination program and remained eligible until they were 80 years old (again, as of September 1 of a given year of the HZ vaccination program); and iii) individuals born on or before September 1 1933 (i.e., aged 80 years and older as of September 1 2013) who never became eligible.

Our analysis was restricted to adults born between September 1 1925 (88 years old on the start date of the HZ vaccination program) and September 1 1942 (71 years old on the start date of the HZ vaccination program). Those born between September 1 1925 and September 1 1933 were never eligible for HZ vaccination, whereas those born between September 2 1933 and September 1 1942 became eligible as part of the catch-up cohort mentioned above. Specifically, the vaccine was offered to those born between: September 2 1933 and September 1 1934 in the first year of the program (September 1 2013 to August 31 2014); September 2 1934 and September 1 1936 in the second year (September 1 2014 to August 31 2015); September 2 1936 and September 1 1937 in the third year (September 1 2015 to August 31 2016); and September 2 1937 and September 1 1938 in the fourth year (September 1 2016 to August 31 2017). As of April 1 2017, individuals became eligible for the vaccine on their 78^th^ birthday and remained eligible until their 80^th^ birthday.

##### Data source and sample size

This study used the Secure Anonymised Information Linkage (SAIL) Databank.^38,85^ This databank provides detailed electronic health record data from primary care in the NHS through the Welsh Longitudinal General Practice dataset,^39^ which contains data on diagnoses, clinical signs and observations, symptoms, laboratory tests and results, procedures performed (including vaccinations), prescribed medications, and administrative items.^86^ Using individuals’ unique NHS number, SAIL links this primary care dataset to a series of databases. For our study, these databases consisted of the Welsh Demographic Service Dataset,^87^ the Patient Episode Database for Wales (containing hospital-based inpatient care data),^88^ the Outpatient Database for Wales (containing specialist-based ambulatory care data),^89^ the Welsh Cancer Intelligence and Surveillance Unit (containing data on care for cancer),^90^ and the Annual District Death Extract (containing cause-of-death data).^91^ Our data included individuals’ date of birth in weeks (with weeks starting on a Monday). A description of each dataset, including the years covered by the data, is provided in Table 2.

We restricted our dataset to all individuals born between September 1 1925 and September 1 1942 who were ever registered with a primary care provider in the Welsh Longitudinal General Practice Dataset and who were alive and residing in Wales as of the start date of the HZ vaccination program (September 1 2013). The resulting sample size was 304,940 individuals, of whom 167,461 were recorded as female, 137,478 as male, and 1 as “unspecified” gender.

### METHOD DETAILS

#### Study cohorts and follow-up period

Given that each patient in our dataset had a unique anonymized NHS number, we were able to follow patients over time even if they changed primary care provider. We defined one study cohort for each of our two aims. For determining the effect of HZ vaccination on the incidence of MCI, we excluded patients whose electronic health record data suggested any cognitive impairment at any time prior to the start date of the HZ vaccination program. To do so, we used the code list for cognitive impairment published by Moran et al. (also shown in Data S1),^97^ which consists of detailed Read codes for any symptoms, signs, and diagnoses relating to cognitive impairment, such as disturbances of memory, orientation, concentration, or reasoning, as well as formal diagnoses of MCI and dementia. For determining the effect of HZ vaccination on the occurrence of deaths due to dementia, we restricted our analysis cohort to those patients with a diagnosis of dementia made at any time prior to the start date of the HZ vaccination program. This cohort is henceforth referred to as patients living with dementia at baseline. The Read and ICD codes used to define dementia (as well as all other diagnoses used in this study) are provided in Data S1.

The follow-up period for all primary analyses was nine years, starting on September 1 2013 (the start date of the HZ vaccination program) and ending on August 31 2022. In secondary analyses, we show all results when using follow-up periods from one to nine years in one-year increments.

#### Exposure and outcome definition

The exposure was eligibility for HZ vaccination based on one’s date of birth. As shown in Figure S10, most eligible patients (especially in the first two eligibility cohorts of the phased rollout, which are the focus of our analysis) in each of our two study populations took up HZ vaccination during their first year of eligibility.

For determining the effect of HZ vaccination on MCI, our primary outcome was MCI as defined by a record of a Read code (see Data S1) for MCI in our electronic health record data. As robustness check, we required that the first diagnosis of MCI not be followed by a new dementia diagnosis within three and within six months to examine the sensitivity of our findings to the possibility of a patient with mild-to-moderate dementia being falsely classified as having MCI. For determining the effect of HZ vaccination on deaths due to dementia, our primary outcome was defined as dementia being named as the underlying (i.e., primary) cause of death in the patient’s death certificate (see Data S1 for ICD-10 codes used). We defined dementia as dementia of any type because of our reduced statistical power when studying less common outcomes, as well as the neuropathological overlap between dementia types and difficulty in distinguishing dementia types clinically.^98–100^ Dates of deaths were for the date of death registration as opposed to occurrence, whereby the median delay between death occurrence and registration in Wales in the years from 2001 to 2021 was five days.^96^

We used all-cause mortality among patients living with dementia at baseline as a secondary outcome. The rationale for analyzing this secondary outcome was that if HZ vaccination reduced deaths due to dementia, it will be important to ascertain whether this effect led to an increase in remaining life expectancy (in which case we would also observe a reduction in all-cause mortality) or merely to the replacement of dementia as the underlying cause of death on the death certificate with the mentioning of another cause (in which case we would observe no effect on all-cause mortality). The Read and ICD codes used to define all our outcomes, including those used as baseline balance checks and in negative control outcome analyses, as well as HZ vaccination are provided in Data S1.

Exploratory outcomes in our analyses were diagnoses of dementia by type. In these analyses, we concentrated on women because our heterogeneous treatment effect analyses indicated that the observed protective effects of HZ vaccination were predominantly driven by this group. We restricted our comparisons to the three most common dementia types in our dataset (Alzheimer’s disease, vascular dementia, and mixed dementia) to avoid overly small sample sizes. We only considered diagnoses made in hospital-based rather than primary care. Hospital-based assessments are more likely to involve comprehensive evaluations, including brain imaging,^47^ thereby likely increasing diagnostic accuracy. Because dementia types differ substantially in incidence, we compared relative rather than absolute effects. Estimating relative effects within a regression discontinuity framework, however, presents methodological constraints, as there is no established approach for calculating confidence intervals or p-values for these estimates.^42^ We therefore report point estimates only. We examined the effect of both HZ vaccination eligibility and receipt on dementia diagnoses by type in two cohorts: i) women without a dementia diagnosis at baseline (i.e., the same cohort as used in Eyting et al.^24^); and ii) women without any record of cognitive impairment at baseline (i.e., the same cohort used for our MCI outcome analysis). We also conducted an analysis focusing on dementia diagnoses that occurred after an MCI diagnosis during the follow-up period. For this analysis, we considered dementia diagnoses made in any care setting to maintain more adequate sample sizes.

#### Ethics

Approval was granted by the Information Governance Review Panel (IGRP, application number: 1306), which oversees and approves applications to use the SAIL databank. This research was also approved by the Stanford University Institutional Review Board on June 9 2023 and considered minimal risk (protocol number: 70277).

### QUANTIFICATION AND STATISTICAL ANALYSIS

The two authors who analyzed the data (M.E. and M.X.) conducted all parts of the analysis independently, compared their results, and, in the case of any discrepancies, agreed on the preferred coding approach through discussion.

#### Regression discontinuity analysis

Patients born immediately before versus immediately after September 2 1933 would be expected to be exchangeable (i.e., similar in observable and unobservable characteristics) with each other except for their probability of receiving HZ vaccination (as a result of their eligibility status for HZ vaccination). Our analysis approach was guided by this expectation.

Regression discontinuity (RD) is a well-established method for causal effect estimation for such threshold-based exposure assignments.^43^ This technique estimates the outcome probability for individuals just on either side of the September 2 1993 date-of-birth eligibility threshold. As per recommended practice for RD,^41,42,82^ we used a mean squared error (MSE)-optimal bandwidth with robust bias-corrected standard errors,^101^ and assigned a higher weight to observations closer to either side of the September 2 1933 date-of-birth eligibility threshold using triangular kernel weights. The MSE-optimal bandwidth was calculated separately for each combination of study cohort and outcome definition. We used local linear regression because it is the recommended and most reliable approach for RD analyses even when the relationship between date of birth and the outcome in the entire dataset is exponential.^102^ However, in robustness checks, we also analyzed our data using local quadratic instead of linear regression. Higher polynomial regressions are not recommended for RD.^102^ In addition, we verified that our results were not dependent on the choice of i) bandwidth (by using bandwidth choices of 0.50, 0.75, 1.25, 1.50, 1.75, and 2.00 times the MSE-optimal bandwidth), and ii) grace period (i.e., the time since the index date after which follow-up time is considered to begin to allow for the time needed for a full immune response to develop after vaccine administration). In an additional robustness check, we adjusted the follow-up period to account for the staggered rollout of the HZ vaccination program. Thus, instead of starting the follow-up period for all individuals on September 1 2013, we started the follow-up period for each individual on the date on which they first became eligible for HZ vaccination. We added cohort fixed effects in these analyses to control for the one- to two-year (depending on the program year) differences between eligibility cohorts in the start of their follow-up period.

For all outcomes, we estimated both the effect of being eligible for HZ vaccination based on one’s date of birth (the intent-to-treat [ITT] effect), as well as the effect of actually receiving HZ vaccination (the complier average causal effect [CACE]). To estimate the CACE, we followed standard practice for RD by implementing a so-called fuzzy RD.^82^ While still comparing individuals just on either side of the date-of-birth eligibility threshold, fuzzy RD corrects the effect estimates for the fact that a proportion of eligible individuals did not receive the vaccine and a small proportion of ineligible individuals did receive the vaccine. Fuzzy RD is implemented by using an instrumental variable approach.^82^ In our analysis, the instrumental variable was a binary indicator for whether or not an individual was eligible for HZ vaccination (i.e., born on or after versus born before September 2 1933). This analysis, therefore, adjusted the effect size of being eligible for HZ vaccination for the magnitude of the abrupt change in the probability of receiving HZ vaccination at the September 2 1933 threshold. Importantly, fuzzy RD does not compare eligible vaccine recipients with eligible vaccine non-recipients because these groups likely have different health characteristics and behaviors (and, thus, confounding is likely).

Given its implementation using local linear regression, RD yields absolute as opposed to relative effect estimates. All regression equations used in our analyses are shown in the last section of the STAR Methods.

#### Testing for confounding

The key advantage of our quasi-randomization approach is that a confounding variable can only bias our analysis if the variable changes abruptly at the September 2 1933 date-of-birth threshold.^41,42^ Thus, confounding bias is unlikely unless another intervention existed that used the identical date-of-birth eligibility threshold (i.e., September 2 1933) as the HZ vaccination program. We investigated whether such a competing intervention was likely to exist in four ways.

First, if another intervention that used the identical date-of-birth eligibility threshold had been implemented prior to the HZ vaccination program, then we may expect to observe differences in patients’ health characteristics or past uptake of preventive health services at the time of the start date of the HZ vaccination program. We, therefore, tested for differences (using the same RD approach as we used for our primary outcomes) across the September 2 1933 date-of-birth threshold in the prevalence of i) diagnoses made at any time prior to September 1 2013 for each of the ten most common causes of disability-adjusted life years (DALYs) and mortality among the age group 70+ years in Wales,^44^ and ii) indicators of past preventive health services uptake. The indicators of past preventive health services uptake available in our data were influenza vaccine receipt in the 12 months preceding program start, receipt of the pneumococcal vaccine as an adult, current statin use (defined as a new or repeat prescription of a statin in the 12 months preceding program start), current use of an antihypertensive medication (defined as a new or repeat prescription of an antihypertensive drug in the 12 months preceding program start), and breast cancer screening participation (defined as the proportion of women with a record of referral to, attendance at, or a report from “breast cancer screening” or mammography at any time prior to the start date of the HZ vaccination program). The codes used to define each of these variables is provided in Data S1.

Second, if a dementia-specific intervention that used the identical date-of-birth eligibility threshold had been implemented before the HZ vaccination program, then we may expect to observe differences in the incidence of our outcomes across the September 2 1933 threshold prior to the start date of the HZ vaccination program. We, thus, conducted the identical analysis as for our primary outcomes except for starting the follow-up period nine years prior to the start date (September 1 2013) of the HZ vaccination program.

Third, if an annual intervention used September 2 as a date-of-birth eligibility criterion, then we may expect to observe significant differences in our outcomes at the September 2 date-of-birth threshold for birth years other than 1933. We, thus, implemented the same analyses as we did for the September 2 1933 threshold for the September 2 threshold of each of the three years of birth preceding and succeeding 1933 (i.e., date-of-birth thresholds of September 2 1930, September 2 1931, September 2 1932, September 2 1934, September 2 1935, and September 2 1936). To ensure that our analyses at these additional date-of-birth thresholds compared individuals of the same age range as in our primary analyses, we shifted the start and end date of the follow-up period to the same extent as the date-of-birth threshold. To maintain the same follow-up period in all comparisons, we, therefore, had to use a follow-up period of six as opposed to nine years. As an example, when comparing individuals across the September 2 1930 threshold, we started the follow-up period on September 1 2010 and ended the follow-up period on August 31 2016.

Fourth, unless another intervention that used the identical date-of-birth eligibility threshold was specifically designed to affect MCI and dementia only, we may expect to see an effect of such an intervention on health outcomes other than MCI, dementia, and deaths due to dementia. We, thus, conducted the same analysis as for our primary outcomes but for diagnoses of, and deaths due to, each of the ten leading causes of DALYs and mortality in Wales for the age group 70+ years,^44^ as well as indicators of preventive health services uptake available in our data. The indicators of preventive health services uptake were breast cancer screening among women (defined as a record of referral to, attendance at, or a report from “breast cancer screening” or mammography at any time after the start date of the HZ vaccination program), and, for the 12 months after the start of the HZ vaccination program, uptake of influenza vaccination as well as any prescription of a statin or antihypertensive medication. Among patients without a record of cognitive impairment prior to the start date of the HZ vaccination program, a new diagnosis of a given condition was defined as the diagnosis being recorded for the first time in our electronic health record data or as an underlying or contributing cause on the death certificate. Among patients with a diagnosis of dementia made prior to the start date of the HZ vaccination program, a death was considered to be caused by a given condition if it was recorded as the underlying cause of death on the death certificate.

#### Testing for ascertainment bias

If healthcare seeking for episodes of shingles constituted an important opportunity for the health system to identify previously undiagnosed MCI, then our analysis for the effect of HZ vaccination on MCI could suffer from ascertainment bias. We conducted three robustness checks to investigate whether this potential ascertainment bias was likely to represent an important source of bias in this study. First, if shingles episodes were an important opportunity for the health system to detect previously undiagnosed chronic conditions, then we may expect to observe an effect of HZ vaccination not only on MCI but also on other common chronic conditions. As described in the preceding section, we, therefore, implemented the same RD analysis as for MCI but for each of the ten leading causes of DALYs and mortality in Wales in 2019 for the age group 70+ years as outcomes.^44^ Second, if healthcare utilization for shingles had an important bearing on the health system’s ability to diagnose MCI, then we may expect that controlling for indicators of healthcare utilization during the follow-up period would attenuate our effect estimates. We, therefore, adjusted our regressions for the number of primary care visits, outpatient visits, hospital admissions, and influenza vaccinations received during our nine-year follow-up period. Third, patients who frequently visit their primary care provider may be more likely to be (whether formally or informally) screened for MCI. An analysis in this cohort of patients should, therefore, be less susceptible to ascertainment bias. We, thus, also implemented our analysis when restricting our study population to the sample of those 135,712 (48.0% of the analysis cohort for our primary analyses for MCI) patients who had made at least one visit to their primary care provider during each of the five years preceding the start of the HZ vaccination program.

#### Evidence triangulation

We used a second quasi-experimental approach, namely a difference-in-differences (DID) analysis, to further investigate the robustness of our RD findings. After restricting our sample to patients born between March 1 1926 and February 28 1934, we implemented our DID approach by dividing our sample into yearly birth cohorts centered around September 1. We then divided each yearly birth cohort into a pre-September birth “season” and a post-September birth season. The pre-September birth season was, thus, defined as the six-months period of March 1 to August 31 and the post-September birth season as the six-months period from September 1 to February 28 of the succeeding year. Our DID model tested whether the difference in outcomes across birth seasons was different for the 1933/1934 birth cohort compared to other yearly birth cohorts. The rationale for our DID was that HZ vaccination eligibility only differed between the two birth seasons in the 1933/1934-cohort but not in other yearly birth cohorts. The DID approach naturally adjusts for any potential systematic differences between pre-September and post-September birth seasons. The regression equations for this DID approach are detailed in the last section of the STAR Methods.

Importantly, our DID did not rely on the continuity assumption (i.e., the assumption that potential confounding variables do not abruptly change at exactly the September 2 1933 date-of-birth eligibility threshold) made by RD. Instead, our DID relied on the assumption that had the HZ vaccination program not existed, then the difference in our outcomes between the pre- and post-September birth seasons would have been the same in the 1933/1934-cohort as in other yearly cohorts. A strength of our approach is that we were able to investigate whether this assumption was likely to be met by testing whether there were significant between-birth-season differences in our outcomes in cohorts other than the 1933/1934-cohort. We did identify such significant differences for MCI among patients without a record of cognitive impairment at baseline, but not for deaths due to dementia among patients living with dementia at baseline. Details are provided in Figure S11. We, therefore, used the DID approach only when analyzing the effect of HZ vaccination on deaths due to dementia.

#### Effect heterogeneity analyses

In our previous analysis for the effect of HZ vaccination on new diagnoses of dementia,^57^ we found a stronger effect among women than men. We, therefore, tested for an effect heterogeneity by gender in both of our aims. To do so, in addition to analyzing the effect among women and men separately, we implemented an interaction model that estimated the difference in effects by gender (the regression equations for this analysis are provided in the last section of the STAR Methods).

We additionally conducted a set of subgroup analyses that aimed to provide suggestive evidence on whether the protective effect for deaths due to dementia also exists among those already living with more advanced dementia at the time of the start date of the HZ vaccination program. We used five measures, all of which were measured during the time period prior to September 1 2013, that we hypothesized are likely correlated with the degree of dementia severity: i) the total number of inpatient days spent in hospital as part of admissions for which dementia was recorded as the principal or secondary reason; ii) the number of hospital admissions for which dementia was recorded as the principal or secondary diagnosis; iii) the number of times that a diagnosis of dementia was recorded in our electronic health record data (regardless of whether the diagnosis was recorded in primary, secondary, or tertiary care); iv) the predicted probability of dying due to dementia (i.e., a death with dementia recorded as the underlying cause on the death certificate) during the follow-up period; and v) the predicted probability of dying from any cause during the follow-up period. We calculated the predicted probability of death from any cause using a logistic regression model with death from any cause during the nine-year follow-up period as outcome and age on September 1 2013 as well as each condition (as measured in the time period prior to September 1 2013) that is part of the Charlson Comorbidity Index (except for AIDS, which is a condition that is suppressed in the SAIL database for privacy reasons) as predictor variables.^46^ The coefficients were first estimated using only patients who were ineligible for HZ vaccination based on their date of birth. The estimated model was then used to predict the risk of death for all patients whilst imputing a fixed age of 80 years. The predicted probability of deaths due to dementia was calculated using the same approach except that we also included the total number of inpatient days spent in hospital (as part of admissions for which dementia was recorded as the principal or secondary reason) and the number of recordings of a diagnosis of dementia as additional predictors. To maximize statistical power for detecting subgroup differences, we defined subgroups for each dementia severity measure based on whether individuals were above or below the median. When using our regression discontinuity approach, we compared both the absolute and relative effects of HZ vaccination on deaths due to dementia between these subgroups. The relative effects were estimated by dividing the absolute effect by the predicted value of the outcome just left (i.e., among those who were ineligible for HZ vaccination when analyzing the effect of vaccination eligibility and among those who were ineligible for HZ vaccination and complied with their eligibility assignment when analyzing the effect of vaccine receipt) of the September 2 1933 date-of-birth eligibility threshold. The confidence intervals and p-values for these relative effect estimates were calculated using the delta method.

#### Regression equations

##### Regression discontinuity

We used the following model to estimate the effect of being *eligible* for HZ vaccination on our outcomes:

(Equation 1) $$Y_{i}=\alpha +\beta_{1}D_{i}+\beta_{2}\cdot \left(WOB_{i}-c_{o}\right)+\beta_{3}D_{i}\cdot \left(WOB_{i}-c_{0}\right)+\epsilon_{i},$$

where $Y_{i}$ was a dichotomous variable indicating if an individual had the outcome, and $D_{i}$ was a dichotomous variable indicating eligibility for HZ vaccination (i.e., $D_{i}$ was equal to one if an individual was born on or after September 2 1933). The term $\left(WOB_{i}\;-\;c_{0}\right)$ and the interaction term $D_{i}\cdot \left(WOB_{i}-c_{0}\right)$ adjusted for how many weeks away from the September 2 1933 date-of-birth eligibility threshold an individual was born. These terms fitted two regression lines on either side of the September 2 1933 date-of-birth eligibility threshold whereby the slope of these regression lines could differ on either side of the threshold. The coefficient $\beta_{1}$ identified the effect of being eligible for HZ vaccination on the outcome.

We used the following model to estimate the effect of *receiving* HZ vaccination on our outcomes:

(Equation 2) $$Y_{i}\;=\;\theta \;+\;\gamma_{1}{\hat{V}}_{i}\;+\;\gamma_{2}\;.\;\left(WOB_{i}\;-\;c_{o}\right)\;+\;\gamma_{3}D_{i}\;.\;\left(WOB_{i}\;-\;c_{0}\right)\;+\;\varepsilon_{i},$$

where $V_{i}$ was a dichotomous variable indicating if an individual received HZ vaccination and was instrumented by $D_{i}.\;{\hat{V}}_{i}$ was the predicted probability of receiving HZ vaccination based on the estimation of Equation 1 with $V_{i}$ as the left-hand-side variable. $\gamma_{1}$ was the effect of receiving HZ vaccination. θ was the constant and $\varepsilon_{i}$ an error term. All other variables were the same as in regression Equation 1. Equation 2, thus, implemented a so-called fuzzy regression discontinuity (RD) design,^82^ which adjusted the effect size for being eligible for HZ vaccination by the size of the abrupt change in the probability of receiving HZ vaccination at the September 2 1933 date-of-birth eligibility threshold. As is common practice for fuzzy RD,^82^ our fuzzy RD achieved this adjustment by using a dichotomous variable that indicated whether or not an individual was eligible to receive HZ vaccination as an instrumental variable for actual receipt of HZ vaccination. For Equations 1 and 2, we restricted the analysis to individuals who were born within the MSE-optimal bandwidths around the threshold, applied triangular kernel weights, and reported p-values and 95% confidence intervals based on robust bias-corrected standard errors.^101^

##### Difference-in-differences

As described in the STAR Methods section in the main manuscript, we implemented our DID approach by dividing our sample into yearly birth cohorts centered around September 1, and then dividing each yearly birth cohort into a pre-September birth “season” (i.e., the six-months period of March 1 to August 31) and a post-September birth season (i.e., the six-months period from September 1 to February 28 of the succeeding year). Our DID approach relied on the assumption that in the absence of the date of birth-based eligibility rule for HZ vaccination, the between-birth-season difference in the outcome would have been the same in the 1933/1934-cohort as in the other yearly birth cohorts. To investigate the validity of this assumption, we estimated the between-birth-season difference in the outcome for each yearly birth cohort, whereby the difference in the 1932/33-cohort served as the reference group.

Specifically, we used the following model:

(Equation 3) $$Y_{i}=\theta +\sum_{h\neq 1932}\left(\gamma^{h}S_{i}\cdot C_{i}^{h}\right)+\eta_{m}+\eta_{c}+\epsilon_{i},$$

where $Y_{i}$ was the outcome of patient i, and $S_{i}$ a dichotomous variable indicating that patient i was born in the post-September birth season. $C_{i}^{h}$ indicated whether patient i belonged to birth cohort h, with h = 1926/27, 1927/28, …, 1931/32, and 1933/34. $\gamma^{h}$ identified the between-birth-season difference of the yearly birth cohort h relative to the 1932/33-cohort. *θ* was the constant term, $\eta_{m}$ and $\eta_{c}$ indicated the birth month and birth cohort fixed-effect, respectively. $\epsilon_{i}$ was the error term. Figure S11 plots the estimates of $\gamma^{h}$ with 95% confidence intervals by yearly birth cohort.

We used a two-stage least squares procedure to implement our DID approach. The first stage estimated the change in the probability of receiving HZ vaccination due to the date of birth-based eligibility rule. We used the following model:

(Equation 4) $$V_{i}=\theta +\gamma S_{i}\cdot C_{i}+\eta_{m}+\eta_{c}+\epsilon_{i},$$

where $V_{i}$ was a dichotomous variable indicating whether patient i had received HZ vaccination, $S_{i}$ a dichotomous variable indicating that patient i was born in the post-September birth season, and $C_{i}$ a dichotomous variable indicating that patient i was in the 1933/1934 birth cohort. γ identified the change in the probability of receiving HZ vaccination as a result of the date of birth-based eligibility rule. θ was a constant term and $\epsilon_{i}$ was the error term. $\eta_{m}$ and $\eta_{c}$ were the birth month and cohort fixed effect, respectively.

In the second stage, we estimated the effect of receipt of HZ vaccination on our outcomes. We used the following model:

(Equation 5) $$Y_{i}=\alpha +\beta {\hat{V}}_{i}+\eta_{m}+\eta_{c}+\varepsilon_{i},$$

where $Y_{i}$ was the outcome of patient i, and ${\hat{V}}_{i}$ was the probability of receiving HZ vaccination as a result of the date of birth-based eligibility rule as estimated from the first-stage regression (4). The coefficient β identified the effect of HZ vaccination receipt on our outcome. θ,$\eta_{m}$, and $\eta_{c}$ were the constant term, birth month, and yearly birth cohort (1926/1927, 1927/1928, …, 1933/1934) fixed effect, respectively. $\varepsilon_{i}$ was the error term.

##### Heterogeneous treatment effect by gender

The difference in CACE by gender was estimated by running the following instrumental variable model:

(Equation 6) $$Y_{i}=\alpha +\beta_{1}V_{i}+\beta_{2}\left(WOB_{i}-c_{0}\right)+\beta_{3}D_{i}\left(WOB_{i}-c_{0}\right)+\beta_{4}V_{i}\cdot MALE_{i}+\beta_{5}\cdot \left(WOB_{i}-c_{0}\right)\cdot MALE_{i}+\beta_{6}D_{i}\cdot \left(WOB_{i}-c_{0}\right)\cdot MALE_{i}\;\;\;+\beta_{7}MALE_{i}+\epsilon_{i}$$

where $Y_{i}$, $D_{i}$, $\left(WOB_{i}-c_{0}\right)$ and $V_{i}$ adopted the same definitions as in Equations 1 and 2. *MALE*_*i*_ was a dichotomous variable indicating if an individual’s gender was male. $\epsilon_{i}$ was the error term. The coefficient $\beta_{4}$ identifies the gender difference in the effect of receipt of the vaccine on the outcome. In estimating the coefficients, $V_{i}$ and $V_{i}\cdot MALE_{i}$ were instrumented by $D_{i}$ and $D_{i}\cdot MALE_{i}$. In line with our primary discontinuity models, we applied triangular weights to the regression along with the MSE-optimal bandwidth sizes from the corresponding specification excluding interaction terms by gender in our primary analyses.

## Supplementary Material

1

2

3

Supplemental information can be found online at https://doi.org/10.1016/j.cell.2025.11.007.

## Figures and Tables

**Table 1.** Characteristics of each dataset used in this study

Dataset	Description

Welsh Demographic Service Dataset (WDSD)^87^	This is a registry of all individuals registered with a primary care provider in Wales (over 98% of adults residing in Wales are registered with a primary care provider^92^). The following variables from this dataset were used in this study: date of birth in weeks, sex, individuals’ anonymized address history (to define residence status in Wales), and the 2011 Welsh Index of Multiple Deprivation (the government’s measure of relative deprivation for small areas in Wales^40^) for each address history. Data were available until March 2023.
Welsh Longitudinal General Practice dataset (WLGP)^39^	This dataset contains comprehensive electronic health record data from primary care for approximately 80% of primary care practices in Wales and 83% of the Welsh population. Our study population was extracted from this dataset. All health events are encoded with Read codes^86^ and include diagnoses, clinical signs and observations, symptoms, medication prescriptions, laboratory tests and results, procedures performed (including vaccinations), and administrative items. This dataset formed the core of our analysis. All variables listed in Data S1 that include Read codes in their definition were at least partially ascertained using this dataset: these variables include those needed to ascertain our study cohorts, primary and secondary outcomes, HZ vaccination, diagnoses for our baseline balance checks and negative control outcome analyses, uptake of preventive health services other than HZ vaccination, and healthcare service utilization indicators. Data were available until January 2023.
Patient Episode Database for Wales (PEDW)^88^	This dataset contains electronic health record data for all inpatient and day case activity undertaken in hospitals of NHS Wales as well as data on Welsh residents treated in hospitals that are part of NHS England. Procedures are encoded using OPCS-4 codes^93^ and diagnoses using ICD-10 codes.^94^ From this dataset, we used dates of admission (to identify the timing of a specific diagnosis and ascertain our healthcare service utilization indicators) and diagnosis codes (to identify a condition first diagnosed in the hospital setting) in this study. The data covered the period from April 1995 to March 2023.
Outpatient Database for Wales (OPDW)^89^	This dataset contains attendance information for all hospital-based outpatient appointments. We used dates of attendances from this dataset to ascertain our healthcare service utilization indicators. Patients in the NHS generally have to obtain a referral from a primary care provider to access outpatient specialist care^84^; thus, referrals to, and diagnoses made in, specialist care are contained in the Welsh Longitudinal General Practice dataset. The data covered the period from January 2004 to March 2023.
Welsh Cancer Intelligence and Surveillance Unit (WCISU)^92^	This is the national cancer registry for Wales, which records all cancer diagnoses given to Welsh residents regardless of the setting in which they were diagnosed or treated. We used dates of diagnoses (to identify the timing of a specific cancer diagnosis) and diagnosis codes (to identify a specific cancer) from this dataset. The data covered the period from January 1994 to July 2022.
Annual District Death Extract (ADDE)^91^	This is the national registry of all deaths of Welsh residents, including deaths of Welsh residents that occurred outside of Wales. Cause-of-death data from death certificates used ICD-9 coding until 2001 and ICD-10 coding thereafter. Dates for deaths were those on which the death was registered, as opposed to when it occurred. The median delay between death occurrence and registration in England and Wales in the years from 2001 to 2021 was 5 days.^95^ We used date of death (to identify the timing of death) as well as cause of death (both underlying and contributing causes) in this study. More detailed information on mortality statistics in the United Kingdom is available elsewhere.^96^ The data covered the period from October 1973 to March 2023.

**Table 2.** Sample characteristics at baseline of the two study cohorts in our analysis

Among patients without cognitive impairment at baseline^a,b,c,d,e^													
		Full sample						Sample in the MSE-optimal bandwidth					
		All		Women		Men		All		Women		Men	
Variable		*n*	%	*n*	%	*n*	%	*n*	%	*n*	%	*n*	%

		282,557	100.0	154,238	54.6	128,318	45.4	58,569	100.0	32,311	55.2	26,258	44.8
Quintile of Welsh Index of Multiple Deprivation	1 (most deprived)	47,321	16.7	26,071	16.9	21,249	16.6	9,654	16.5	5,391	16.7	4,263	16.2
	2	53,628	19.0	29,517	19.1	24,111	18.8	11,060	18.9	6,248	19.3	4,812	18.3
	3	61,528	21.8	33,596	21.8	27,932	21.8	12,854	21.9	7,081	21.9	5,773	22.0
	4	58,352	20.7	31,461	20.4	26,891	21.0	12,117	20.7	6,569	20.3	5,548	21.1
	5 (least deprived)	61,728	21.8	33,593	21.8	28,135	21.9	12,884	22.0	7,022	21.7	5,862	22.3
Clinical diagnoses	past shingles	34,280	12.1	20,461	13.3	13,819	10.8	7,611	13.0	4,592	14.2	3,019	11.5
	ischemic heart disease	45,456	16.1	17,928	11.6	27,528	21.5	10,488	17.9	4,168	12.9	6,320	24.1
	COPD	33,191	11.7	15,586	10.1	17,605	13.7	7,164	12.2	3,314	10.3	3,850	14.7
	past stroke	20,938	7.4	10,028	6.5	10,910	8.5	4,879	8.3	2,362	7.3	2,517	9.6
	past lower respiratory tract infection	145,299	51.4	80,215	52.0	65,084	50.7	30,880	52.7	16,980	52.6	13,900	52.9
	history of lung cancer	1,179	0.4	554	0.4	625	0.5	268	0.5	130	0.4	138	0.5
	past fall(s)	52,620	18.6	35,998	23.3	16,622	13.0	11,897	20.3	8,161	25.3	3,736	14.2
	history of colorectal cancer	5,662	2.0	2,445	1.6	3,217	2.5	1,295	2.2	557	1.7	738	2.8
	history of lower back pain	126,210	44.7	72,251	46.8	53,959	42.1	26,471	45.2	15,379	47.6	11,092	42.2
	history of breast cancer	–	–	6,450	4.2	–	–	–	–	1,319	4.1	–	–
	history of pancreatic cancer	162	<0.1	81	<0.1	81	<0.1	28	<0.1	14	<0.1	14	<0.1
	diabetes mellitus	55,354	19.6	26,289	17.0	29,065	22.7	12,185	20.8	5,903	18.3	6,282	23.9
Uptake of preventive health measures	breast cancer screening	–	–	34,680	22.5	–	–	–	–	5,952	18.4	–	–
	PPV-23	198,677	70.3	106,803	69.2	91,874	71.6	42,872	73.2	23,109	71.5	19,763	75.3
	influenza vaccine	193,856	68.6	103,677	67.2	90,178	70.3	41,411	70.7	22,247	68.9	19,164	73.0
	recent statin use	130,175	46.1	65,910	42.7	64,265	50.1	27,747	47.4	14,449	44.7	13,298	50.6
	recent antihypertensive use	172,148	60.9	93,026	60.3	79,122	61.7	37,711	64.4	20,651	63.9	17,060	65.0
Among patients living with dementia at baseline^a,b,c,d,e^													
		Full sample						Sample in the MSE-optimal bandwidth					
		All		Women		Men		All		Women		Men	
Variable		*n*	%	*n*	%	*n*	%	*n*	%	*n*	%	*n*	%

		14,350	100.0	8,957	62.4	5,393	37.6	3,418	100.0	2,064	60.4	1,354	39.6
Quintile of Welsh Index of Multiple Deprivation	1 (most deprived)	2,734	19.1	1,699	19.0	1,035	19.2	648	19.0	408	19.8	240	17.7
	2	3,012	21.0	1,897	21.2	1,115	20.7	729	21.3	458	22.2	271	20.0
	3	3,021	21.1	1,932	21.6	1,089	20.2	730	21.4	440	21.3	290	21.4
	4	2,788	19.4	1,718	19.2	1,070	19.8	655	19.2	379	18.4	276	20.4
	5 (least deprived)	2,795	19.5	1,711	19.1	1,084	20.1	656	19.2	379	18.4	277	20.5
Clinical diagnoses	past shingles	1,761	12.3	1,176	13.1	585	10.8	400	11.7	254	12.3	146	10.8
	ischemic heart disease	2,975	20.7	1,513	16.9	1,462	27.1	725	21.2	360	17.4	365	27.0
	COPD	2,073	14.4	1,133	12.6	940	17.4	475	13.9	253	12.3	222	16.4
	past stroke	2,654	18.5	1,461	16.3	1,193	22.1	640	18.7	325	15.7	315	23.3
	past lower respiratory tract infection	8,661	60.4	5,365	59.9	3,296	61.1	2,013	58.9	1,196	57.9	817	60.3
	history of lung cancer	58	0.4	28	0.3	30	0.6	20	0.6	10	0.5	10	0.7
	past fall(s)	6,652	46.4	4,608	51.4	2,044	37.9	1,476	43.2	983	47.6	493	36.4
	history of colorectal cancer	285	2.0	138	1.5	147	2.7	60	1.8	25	1.2	35	2.6
	history of lower back pain	6,530	45.5	4,161	46.5	2,369	43.9	1,573	46.0	996	48.3	577	42.6
	history of breast cancer	–	–	298	3.3	–	–	–	–	68	3.3	–	–
	history of pancreatic cancer	<10	<0.1	<10	<0.1	<10	<0.1	<10	<0.1	<10	<0.1	<10	<0.1
	diabetes mellitus	3,332	23.2	1,926	21.5	1,406	26.1	853	25.0	486	23.5	367	27.1
Uptake of preventive health measures	breast cancer screening	–	–	1,327	14.8	–	–	–	–	323	15.6	–	–
	PPV-23	10,478	73.0	6,330	70.7	4,148	76.9	2,512	73.5	1,473	71.4	1,039	76.7
	influenza vaccine	9,586	66.8	5,840	65.2	3,746	69.5	2,263	66.2	1,331	64.5	932	68.8
	recent statin use	6,596	46.0	3,863	43.1	2,733	50.7	1,650	48.3	938	45.4	712	52.6
	recent antihypertensive use	7,535	52.5	4,608	51.4	2,927	54.3	1,801	52.7	1,058	51.3	743	54.9

Abbreviations are as follows: Sept, September; MSE, mean squared error; COPD, chronic obstructive pulmonary disease; PPV-23, pneumococcal polysaccharide vaccine.

a The baseline date was September 1, 2013 (the start date of the HZ vaccination program).

b The length of the MSE-optimal bandwidth (used in our complier average causal effect analysis) was 95.1 weeks for patients without cognitive impairment at baseline and 97.5 weeks for patients living with dementia at baseline.

c Deciles of the Welsh Index of Multiple Deprivation (WIMD) were calculated based on the 2011 WIMD survey.^40^

d Breast cancer screening was defined as the proportion of women with a record of referral to, attendance at, or a report from “breast cancer screening” or mammography at any time prior to September 1, 2013. “PPV-23” was defined as receipt of the pneumococcal vaccine as an adult at any time prior to September 1, 2013. “Influenza vaccine” was defined as influenza vaccine receipt in the 12 months preceding September 1, 2013. Recent statin and antihypertensive use was defined as a new or repeat prescription of a statin or antihypertensive drug, respectively, in the 12 months preceding September 1, 2013.

e Diabetes mellitus referred to both diabetes mellitus type 1 and type 2.

**Table:** KEY RESOURCES TABLE

REAGENT or RESOURCE	SOURCE	IDENTIFIER

Deposited data		

Welsh Demographic Service Dataset (WDSD)	SAIL databank	ID: 1306
Welsh Longitudinal General Practice dataset (WLGP)	SAIL databank	ID: 1306
Patient Episode Database for Wales (PEDW)	SAIL databank	ID: 1306
Outpatient Database for Wales (OPDW)	SAIL databank	ID: 1306
Annual District Death Extract (ADDE)	SAIL databank	ID: 1306
Welsh Cancer Intelligence and Surveillance Unit (WCISU)	SAIL databank	ID: 1306

Software and algorithms		

R 4.3.3	R Core Team	http://r-project.org/
Omnissa Horizon Client 2503	Omnissa	https://www.omnissa.com/products/horizon-8/
DB2 11.1	IBM	https://www.ibm.com/products/db2

## Highlights

- Herpes zoster vaccination reduced dementia diagnosis in our prior natural experiments
- Here, we find a lower occurrence of MCI and dementia deaths among dementia patients
- Herpes zoster vaccination appears to act along the entire clinical course of dementia
- This study’s approach avoids the common confounding concerns of observational data
