# Supplementary material for: The effect of shingles vaccination at different stages of the dementia disease course
Source: Cell. Author manuscript; Available in PMC 2026 Jan 29. (PMC12851904; doi:10.1016/j.cell.2025.11.007)
Supplement: 1 [file NIHMS2122152-supplement-1.pdf]

Cell, Volume *188*

Supplemental information

The effect of shingles vaccination at different stages of the dementia disease course

Min Xie, Markus Eyting, Christian Bommer, Haroon Ahmed, and Pascal Geldsetzer

	Mild cognitive impairment	Deaths due to dementia	All-cause mortality
Difference in CACE by gender	4.9	55.9	30.2
95% CI	(0.5, 9.3)	(2.8, 109.0)	(-0.9, 61.3)
p	0.029	0.039	0.057
Bandwidth (in weeks)	95.1	97.5	106.4
Observations	58,569	3,418	3,748

Table S1. Difference (in percentage points) between women and men in the effect of receipt of HZ vaccination on new diagnoses of mild cognitive impairment, deaths due to dementia and all-cause mortality, related to Figure 4 and Figure S8.[1]

[1] The CACE (complier average causal effect) refers to the estimated effect of actually receiving HZ vaccination as opposed to merely being eligible for vaccination. The reference group was women when calculating the difference in CACE. Bandwidth refers to the mean squared error-optimal bandwidth. Observations refer to the number of observations within the mean squared error-optimal bandwidth.

Abbreviations: CACE = complier average causal effect; CI = confidence interval; p = p-value

Sample as defined at baseline[1]	Outcome	Type of dementia		
		Vascular dementia	***Alzheimer's disease***	***Mixed dementia***
Effect of vaccine receipt (%)				
Without a dementia diagnosis[2]	Dementia diagnosis	-12.3	-15.7	-30.6
Without cognitive impairment	Dementia diagnosis	-10.5	-16.5	-27.5
	Dementia diagnosis subsequent to MCI diagnosis[3]	-30.8	-29.7	-41.1
Effect of vaccination eligibility (%)				
Without a dementia diagnosis[2]	Dementia diagnosis	-4.5	-7.6	-30.6
Without cognitive impairment	Dementia diagnosis	-3.7	-8.4	-14.9
	Dementia diagnosis subsequent to MCI diagnosis[3]	-22.5	-18.6	-21.8

Table S2. The relative effect of HZ vaccination on new diagnoses of dementia among women by dementia type, related to Figure 4.

[1] Baseline refers to the start date (September 1 2013) of the HZ vaccination program.
[2] This is the same sample as used in Eyting et al. 2025.
[3] This row shows the relative effect of HZ vaccination among individuals without cognitive impairment at baseline and who received a dementia diagnosis after their MCI diagnosis at any time during the follow-up period, separately for each type of dementia.
Abbreviations: MCI = mild cognitive impairment
