# Supplementary material for: The effect of shingles vaccination at different stages of the dementia disease course
Source: Cell. Author manuscript; Available in PMC 2026 Jan 29. (PMC12851904; doi:10.1016/j.cell.2025.11.007)
Supplement: 3 [file NIHMS2122152-supplement-3.pdf]

Supplemental figures

Among patients without cognitive impairment at baseline

	Effect size, 95% CI (percentage points)	p
**Leading causes of morbidity and mortality**		
Stroke	−0.2 (−1.2, 0.8)	0.645
Shingles	0.1 (−1.2, 1.4)	0.887
Pancreatic cancer	0.0 (−0.1, 0.1)	0.926
Mild cognitive impairment	−0.2 (−1.1, 0.4)	0.390
Lung cancer	0.1 (−0.2, 0.3)	0.493
Lower respiratory tract infection	0.2 (−1.7, 1.9)	0.909
Lower back pain	−0.9 (−3.0, 0.9)	0.299
Ischemic heart disease	−0.7 (−2.2, 0.4)	0.173
Falls	0.4 (−0.9, 2.0)	0.462
Diabetes mellitus	0.0 (−1.5, 1.5)	0.999
Dementia	0.5 (−0.3, 1.4)	0.174
Colorectal cancer	0.4 (−0.1, 1.2)	0.113
COPD	0.0 (−1.1, 1.3)	0.933
Breast cancer	0.2 (−0.7, 1.0)	0.765
**Indicators of preventive health services uptake**		
Statin use	0.8 (−0.7, 2.7)	0.244
PPV vaccination	−0.1 (−2.0, 1.3)	0.696
Influenza vaccination	0.0 (−1.5, 1.4)	0.918
Breast cancer screening	−0.5 (−2.5, 1.5)	0.626
Antihypertensive medication use	0.7 (−1.1, 2.5)	0.455

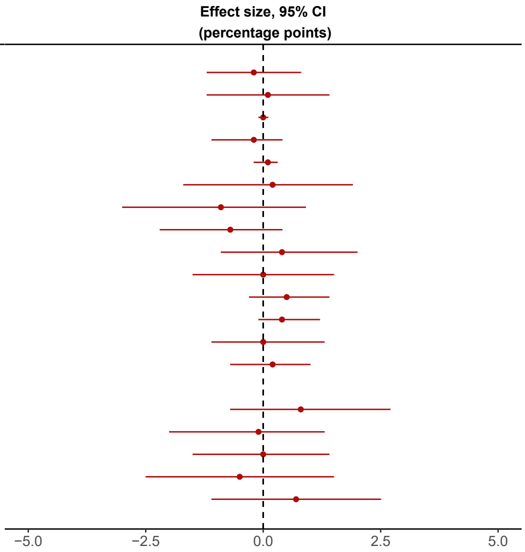

Among patients living with dementia at baseline

	Effect size, 95% CI (percentage points)	p
**Leading causes of morbidity and mortality**		
Stroke	0.8 (−4.8, 7.3)	0.691
Pancreatic cancer	0.2 (−0.1, 0.5)	0.173
Lung cancer	0.5 (−0.2, 1.3)	0.130
Lower respiratory tract infection	−4.3 (−12.1, 1.8)	0.148
Lower back pain	1.2 (−6.8, 10.5)	0.680
Ischemic heart disease	−8.5 (−15.8, −2.4)	0.008
Falls	−3.1 (−10.4, 4.4)	0.424
Diabetes mellitus	−0.6 (−7.2, 6.8)	0.960
Deaths due to dementia	−5.2 (−17.7, 6.7)	0.375
Colorectal cancer	−0.3 (−2.2, 1.8)	0.859
COPD	0.4 (−4.5, 5.1)	0.888
Breast cancer	2.3 (−0.7, 6.3)	0.121
**Indicators of preventive health services uptake**		
Statin use	−1.9 (−9.8, 5.2)	0.547
PPV vaccination	−1.2 (−7.3, 4.5)	0.633
Influenza vaccination	−0.4 (−7.8, 7.0)	0.916
Breast cancer screening	6.5 (−0.8, 16.8)	0.076
Antihypertensive medication use	3.2 (−5.2, 11.8)	0.447

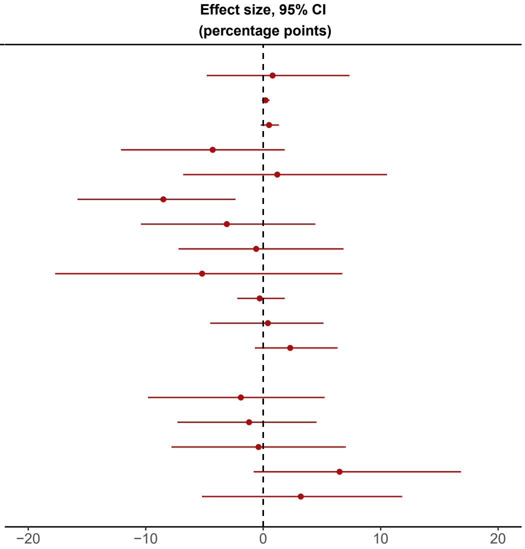

Figure S1. Baseline balance checks using diagnoses of the ten most common causes of DALYs and mortality among adults aged 70+ years in Wales, as well as indicators of preventive health services uptake, related to Figure 1

Baseline refers to the start date of the HZ vaccination program (i.e., September 1, 2013). We used diagnoses of the ten most common causes of disability-adjusted life years (DALYs) and mortality among the age group 70+ years in Wales, as estimated by the 2019 Global Burden of Disease study.[44] Statin use was defined as a new or repeat prescription of a statin in the 12 months preceding September 1, 2013. PPV vaccination was defined as receipt of the PPV vaccine as an adult at any time prior to September 1, 2013. Influenza vaccination was defined as receipt of the influenza vaccine in the 12 months preceding September 1, 2013. Breast cancer screening was defined as the proportion of women with a record of referral to, attendance at, or a report from breast cancer screening or mammography at any time prior to September 1, 2013. Antihypertensive medication use was defined as a new or repeat prescription of an antihypertensive drug in the 12 months preceding September 1, 2013. The study cohorts for the baseline balance checks for MCI and dementia did not exclude individuals with any record of cognitive impairment prior to September 1, 2013. Similarly, the study cohort for the baseline balance check for deaths due to dementia was not restricted to individuals who were alive on September 1, 2013. For all other baseline balance checks, the study cohort was defined as detailed in the STAR Methods. Dots show the point estimate and horizontal bars the 95% CI. The codes used to define each condition are listed in Data S1.

Abbreviations are as follows: CI, confidence interval; COPD, chronic obstructive pulmonary disease; PPV, pneumococcal polysaccharide vaccine.

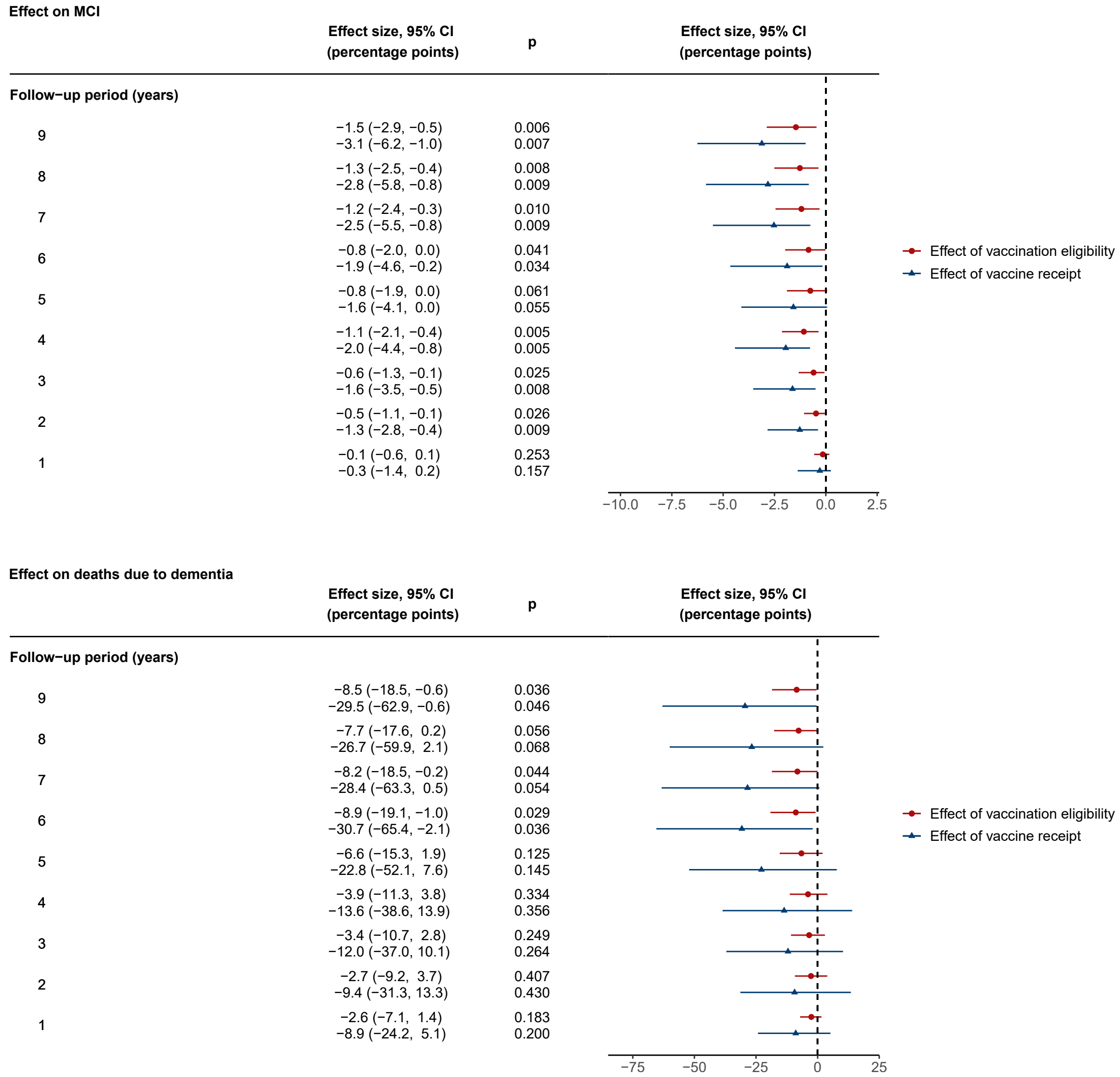

Figure S2. The effect of HZ vaccination eligibility and receipt on our outcomes across different lengths of follow-up, related to Figure 2

Dots or triangles show the point estimate and horizontal bars the 95% CI.

Abbreviations are as follows: MCI, mild cognitive impairment; CI, confidence interval.

Effect on MCI

	Effect size, 95% CI (percentage points)	p
**Bandwidth choice (as x−times the MSE−optimal bandwidth)**		
2	−0.9 (−1.7, −0.1)	0.033
	−1.9 (−4.0, −0.3)	0.026
1.75	−1.0 (−2.0, −0.2)	0.017
	−2.1 (−4.5, −0.5)	0.014
1.5	−1.1 (−2.3, −0.3)	0.009
	−2.4 (−4.9, −0.7)	0.011
1.25	−1.4 (−2.6, −0.5)	0.005
	−2.9 (−5.7, −1.0)	0.005
1	−1.5 (−2.9, −0.5)	0.006
	−3.1 (−6.2, −1.0)	0.007
0.75	−1.4 (−2.9, −0.2)	0.027
	−3.1 (−6.5, −0.2)	0.036
0.5	−1.7 (−3.4, 0.0)	0.056
	−3.7 (−8.3, −0.1)	0.046
**Grace−period (months)**		
4	−1.5 (−3.0, −0.5)	0.005
	−3.3 (−6.5, −1.2)	0.004
2	−1.5 (−2.9, −0.5)	0.006
	−3.1 (−6.3, −1.0)	0.007
0	−1.5 (−2.9, −0.5)	0.006
	−3.1 (−6.2, −1.0)	0.007
**Alternative outcome definition**		
MCI not followed by dementia within 6 months	−1.3 (−2.6, −0.4)	0.006
	−2.8 (−5.6, −1.0)	0.004
MCI not followed by dementia within 3 months	−1.4 (−2.8, −0.5)	0.006
	−3.0 (−5.9, −1.1)	0.005
**Other checks**		
Restricting to frequent GP visitors	−1.3 (−2.8, −0.3)	0.017
	−2.6 (−5.7, −0.4)	0.022
Quadratic fit	−1.7 (−3.3, −0.6)	0.006
	−3.5 (−6.7, −1.1)	0.007
Controlling for health service utilization	−1.5 (−2.8, −0.5)	0.006
	−3.1 (−6.1, −1.0)	0.006
Accounting for staggered roll−out	−1.5 (−2.8, −0.5)	0.005
	−3.3 (−6.8, −0.5)	0.022

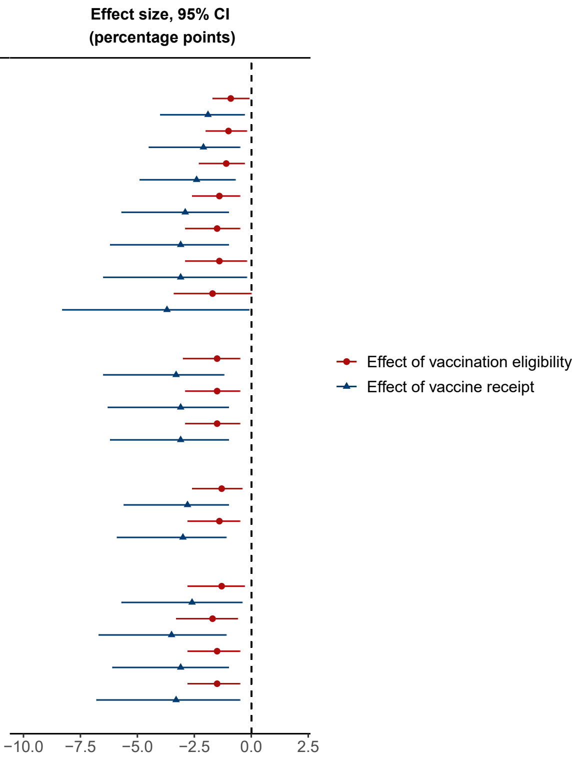

Effect on deaths due to dementia

	Effect size, 95% CI (percentage points)	p
**Bandwidth choice (as x−times the MSE−optimal bandwidth)**		
2	−5.4 (−12.9, −0.4)	0.036
	−20.6 (−50.1, −2.0)	0.033
1.75	−5.8 (−13.9, −0.5)	0.034
	−22.0 (−53.1, −2.2)	0.033
1.5	−6.4 (−14.7, −0.2)	0.043
	−23.5 (−54.2, −0.4)	0.047
1.25	−7.0 (−15.9, 0.0)	0.051
	−24.7 (−55.5, 1.0)	0.059
1	−8.5 (−18.5, −0.6)	0.036
	−29.5 (−62.9, −0.6)	0.046
0.75	−9.0 (−20.5, 0.2)	0.055
	−32.2 (−73.1, 1.5)	0.060
0.5	−8.4 (−21.1, 4.7)	0.212
	−29.3 (−73.9, 16.6)	0.215
**Grace−period (months)**		
4	−8.0 (−17.8, −0.1)	0.048
	−27.9 (−60.7, 1.4)	0.061
2	−8.1 (−17.8, −0.2)	0.045
	−28.0 (−60.7, 1.0)	0.058
0	−8.5 (−18.5, −0.6)	0.036
	−29.5 (−62.9, −0.6)	0.046
**Other checks**		
Quadratic fit	−8.9 (−19.6, 0.4)	0.061
	−29.6 (−64.6, 2.7)	0.072
Adjusting for baseline IHD diagnosis	−7.7 (−13.7, −1.7)	0.012
	−27.1 (−48.2, −6.0)	0.012
Accounting for staggered roll−out	−8.1 (−18.0, −0.3)	0.042
	−31.3 (−69.2, −0.2)	0.048

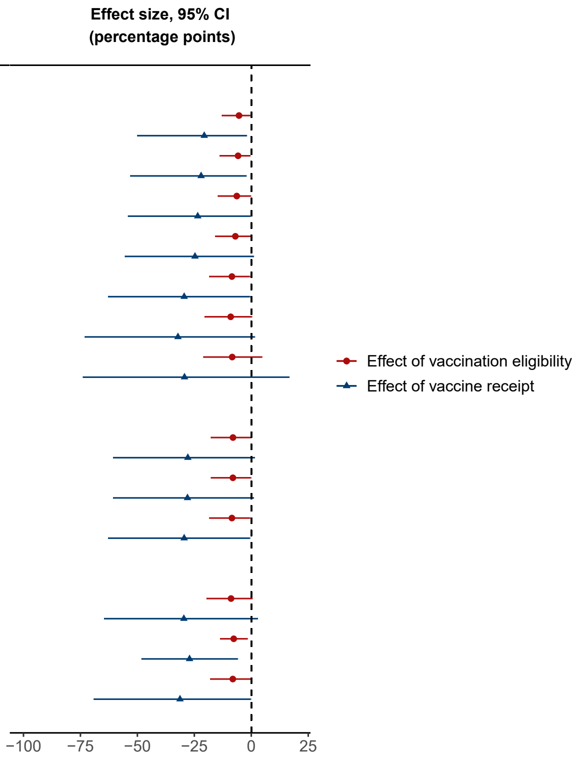

(legend on next page)

Figure S3. Robustness checks for the effect of HZ vaccination eligibility and receipt on our outcomes, related to Figure 2

Dots or triangles show the point estimate and horizontal bars the 95% CI. With "grace periods," we refer to time periods since the index date after which follow-up time is considered to begin to allow for the time needed for a full immune response to develop after vaccine administration. Frequent GP visitors were defined as patients who had made at least one visit to their primary care provider during each of the 5 years preceding the start of the HZ vaccination program. The health service utilization indicators that were used when controlling for health service utilization were the number of primary care visits, outpatient visits, hospital admissions, and influenza vaccinations received during our 9-year follow-up period. As described in detail in the STAR Methods, when accounting for the staggered roll-out of the program, we adjusted the follow-up period to begin for each individual on the date on which they first became eligible for HZ vaccination (instead of starting the follow-up period for all individuals on September 1, 2013). We added cohort fixed effects to these analyses to control for between-cohort differences in the date at which the follow-up window started. That is, we defined one cohort fixed effect for ineligible individuals and the first catch-up cohort and included additional cohort fixed effects for each group of patients who became eligible at the same time.

Abbreviations are as follows: MCI, mild cognitive impairment; CI, confidence interval; MSE, mean squared error; GP, general practitioner; IHD, ischemic heart disease.

	Effect size, 95% CI (percentage points)	p
**Eligibility cutoff with a 6−year follow−up**		
1936−09−02	0.3 (−0.5, 1.4)	0.363
	0.2 (−7.5, 8.5)	0.902
1935−09−02	−0.2 (−1.0, 0.7)	0.721
	0.8 (−7.8, 8.1)	0.965
1934−09−02	0.2 (−0.6, 1.2)	0.532
	−0.7 (−7.4, 5.6)	0.783
1933−09−02	−0.8 (−2.0, 0.0)	0.041
	−8.8 (−19.1, −1.0)	0.029
1932−09−02	0.4 (−0.5, 1.3)	0.345
	3.8 (−4.9, 11.9)	0.412
1931−09−02	0.1 (−0.9, 1.3)	0.704
	3.4 (−4.9, 11.8)	0.424
1930−09−02	−0.6 (−2.2, 0.6)	0.252
	2.2 (−5.4, 10.4)	0.537

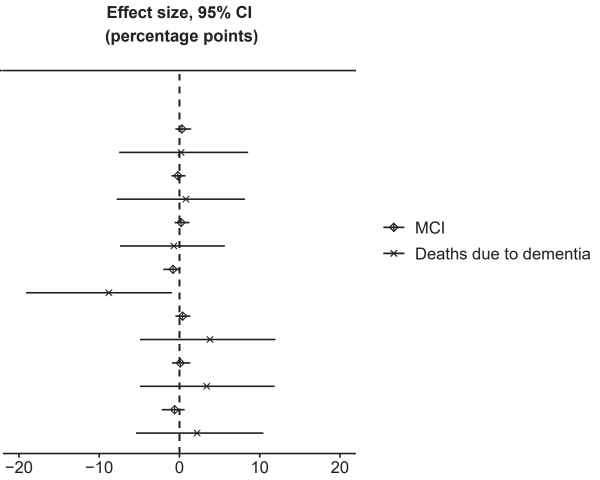

Figure S4. The September 2 date-of-birth threshold only had a significant effect on our outcomes in the birth year (1933) that was used by the herpes zoster vaccination program as eligibility criterion, related to Figure 2

Diamonds or crosses show the point estimate and horizontal bars the 95% CI. New diagnoses of MCI were analyzed among a study cohort of patients who did not have any record of cognitive impairment prior to the start of the follow-up period. Deaths due to dementia were analyzed among a study cohort of patients who had received a diagnosis of dementia prior to the start of the follow-up period. We used a 6-year instead of a 9-year follow-up period in this analysis to allow for the same length of follow-up for all comparisons.

Abbreviations are as follows: CI, confidence interval; MCI, mild cognitive impairment.

Effect on MCI among women

	Effect size, 95% CI (percentage points)	p
**Bandwidth choice (as x−times the MSE−optimal bandwidth)**		
2	−1.5 (−2.9, −0.5)	0.004
	−2.5 (−6.1, −0.9)	0.008
1.75	−1.7 (−3.3, −0.8)	0.002
	−3.1 (−7.1, −1.5)	0.003
1.5	−2.0 (−3.7, −1.0)	<0.001
	−3.6 (−8.0, −2.0)	0.001
1.25	−2.3 (−4.1, −1.1)	<0.001
	−4.3 (−8.8, −2.3)	<0.001
1	−2.5 (−4.5, −1.1)	0.001
	−5.1 (−9.8, −2.6)	<0.001
0.75	−2.6 (−4.8, −0.9)	0.004
	−5.6 (−10.7, −2.3)	0.003
0.5	−2.7 (−5.2, −0.3)	0.026
	−5.9 (−11.9, −1.0)	0.021
**Grace−period (months)**		
4	−2.5 (−4.6, −1.1)	0.001
	−5.3 (−10.1, −2.7)	<0.001
2	−2.4 (−4.4, −1.0)	0.002
	−4.9 (−9.6, −2.3)	0.001
0	−2.5 (−4.5, −1.1)	0.001
	−5.1 (−9.8, −2.6)	<0.001
**Alternative outcome definition**		
MCI not followed by dementia within 6 months	−2.4 (−4.2, −1.1)	<0.001
	−4.8 (−9.1, −2.6)	<0.001
MCI not followed by dementia within 3 months	−2.5 (−4.5, −1.2)	<0.001
	−5.1 (−9.6, −2.8)	<0.001
**Other checks**		
Restricting to frequent GP visitors	−2.4 (−4.5, −0.9)	0.004
	−4.3 (−9.0, −1.8)	0.004
Quadratic fit	−2.8 (−4.8, −1.2)	0.001
	−6.1 (−10.6, −2.5)	0.001
Controlling for health service utilization	−2.4 (−4.3, −1.2)	<0.001
	−5.1 (−9.7, −2.6)	<0.001
Accounting for staggered roll−out	−2.4 (−4.4, −1.1)	0.001
	−5.4 (−10.3, −2.5)	0.001

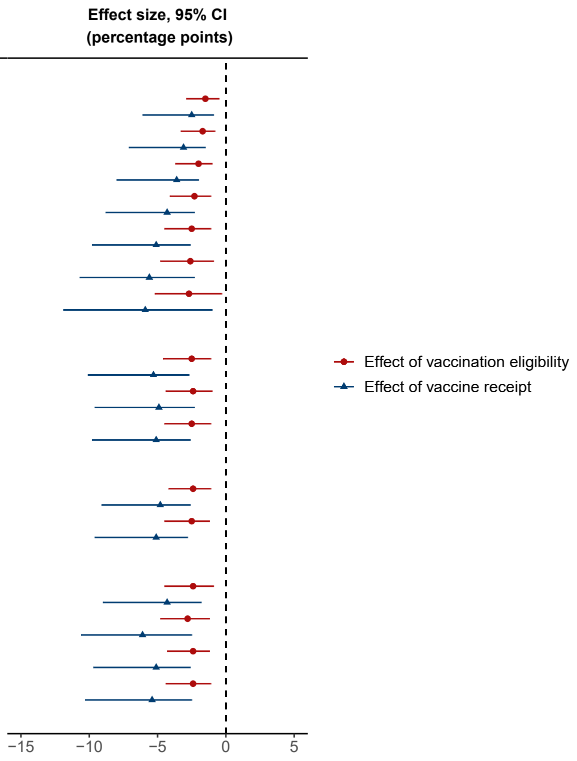

Effect on deaths due to dementia among women

	Effect size, 95% CI (percentage points)	p
**Bandwidth choice (as x−times the MSE−optimal bandwidth)**		
2	−10.9 (−20.2, −4.3)	0.002
	−45.4 (−85.0, −17.0)	0.003
1.75	−11.3 (−21.5, −4.5)	0.003
	−46.4 (−88.7, −17.1)	0.004
1.5	−11.6 (−22.2, −3.8)	0.006
	−46.5 (−89.0, −13.1)	0.008
1.25	−11.8 (−22.8, −2.5)	0.015
	−45.5 (−86.7, −7.0)	0.021
1	−13.9 (−26.3, −3.4)	0.011
	−52.3 (−97.9, −9.2)	0.018
0.75	−15.2 (−30.4, −3.8)	0.012
	−57.9 (−117.0, −11.7)	0.017
0.5	−14.2 (−30.2, 2.8)	0.103
	−52.3 (−111.9, 13.1)	0.121
**Grace−period (months)**		
4	−13.7 (−25.7, −3.4)	0.010
	−52.0 (−96.7, −8.7)	0.019
2	−14.5 (−26.7, −4.2)	0.007
	−55.0 (−100.3, −11.5)	0.014
0	−13.9 (−26.3, −3.4)	0.011
	−52.3 (−97.9, −9.2)	0.018
**Other checks**		
Quadratic fit	−14.0 (−27.5, −1.8)	0.026
	−50.5 (−98.6, −3.4)	0.036
Adjusting for baseline IHD diagnosis	−14.4 (−23.7, −5.1)	0.002
	−60.7 (−98.8, −22.6)	0.002
Accounting for staggered roll−out	−12.7 (−24.8, −2.3)	0.018
	−51.7 (−100.5, −6.1)	0.027

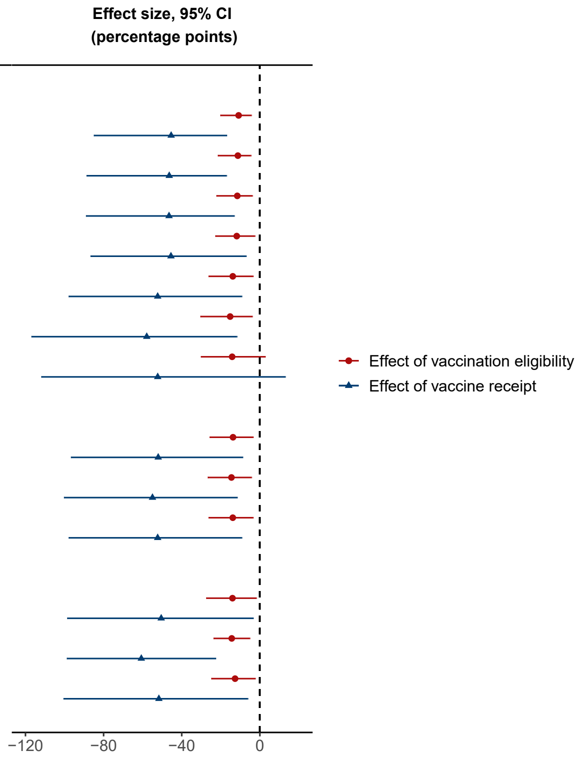

(legend on next page)

Figure S5. Robustness checks for the effect of HZ vaccination eligibility and receipt on our outcomes among women only, related to Figure 4

Dots or triangles show the point estimate and horizontal bars the 95% CI. With grace periods, we refer to time periods since the index date after which follow-up time is considered to begin to allow for the time needed for a full immune response to develop after vaccine administration. Frequent GP visitors were defined as patients who had made at least one visit to their primary care provider during each of the 5 years preceding the start of the HZ vaccination program. The health service utilization indicators that were used when controlling for health service utilization were the number of primary care visits, outpatient visits, hospital admissions, and influenza vaccinations received during our 9-year follow-up period. As described in detail in the STAR Methods, when accounting for the staggered roll-out of the program, we adjusted the follow-up period to begin for each individual on the date on which they first became eligible for HZ vaccination (instead of starting the follow-up period for all individuals on September 1, 2013). We added cohort fixed effects to these analyses to control for between-cohort differences in the date at which the follow-up window started. That is, we defined one cohort fixed effect for ineligible individuals and the first catch-up cohort and included additional cohort fixed effects for each group of patients who became eligible at the same time.

Abbreviations are as follows: MCI, mild cognitive impairment; CI, confidence interval; MSE, mean squared error; GP, general practitioner; IHD, ischemic heart disease.

Dementia severity measure	N	Effect size, 95% CI (percentage points)	p
**Predicted risk of death due to dementia**			
Effect of vaccine receipt	2008	−20.7 (−61.6, 18.8)	0.296
	1706	−36.0 (−84.4, 4.1)	0.076
Effect of vaccination eligibility	2008	−6.2 (−18.5, 5.1)	0.268
	1706	−10.0 (−24.1, 0.8)	0.067
**Predicted risk of all−cause mortality**			
Effect of vaccine receipt	2306	−23.2 (−61.0, 14.5)	0.227
	1587	−35.6 (−86.9, 8.1)	0.104
Effect of vaccination eligibility	2306	−6.7 (−17.8, 3.9)	0.212
	1587	−10.1 (−24.8, 1.6)	0.084
**Length of dementia−related hospital stay**			
Effect of vaccine receipt	2131	−9.1 (−40.0, 23.3)	0.606
	1615	−70.2 (−152.8, −5.6)	0.035
Effect of vaccination eligibility	2131	−3.3 (−14.8, 8.4)	0.587
	1615	−13.5 (−28.8, −2.3)	0.022
**Number of dementia−related hospital admissions**			
Effect of vaccine receipt	2665	−22.8 (−55.1, 5.4)	0.108
	1073	−50.7 (−189.0, 77.3)	0.411
Effect of vaccination eligibility	2665	−7.7 (−18.8, 1.5)	0.093
	1073	−6.7 (−24.6, 9.4)	0.378
**Number of recordings of dementia**			
Effect of vaccine receipt	2292	−23.0 (−61.9, 10.0)	0.157
	1478	−33.7 (−92.0, 19.3)	0.200
Effect of vaccination eligibility	2292	−7.1 (−19.4, 2.8)	0.141
	1478	−8.3 (−23.3, 4.1)	0.171

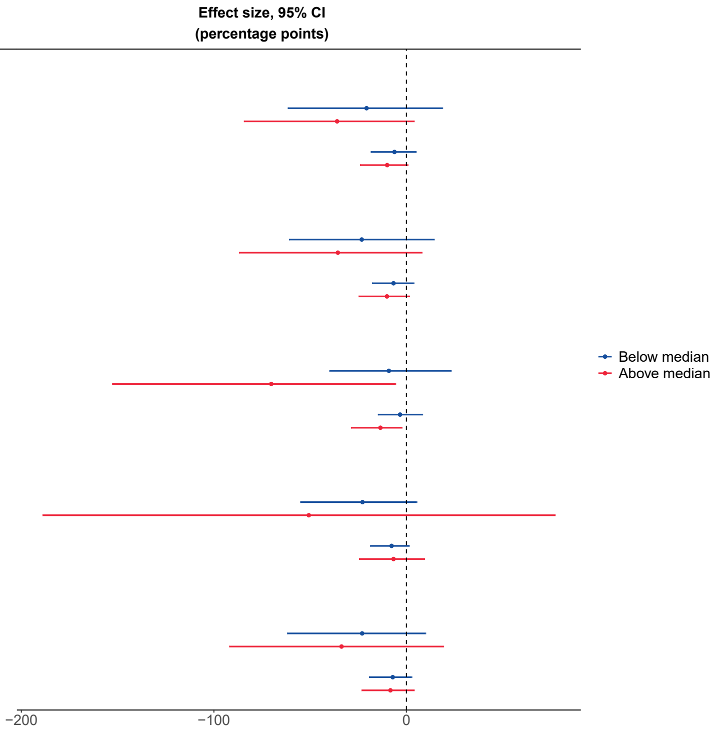

Dementia severity measure	N	Effect size, 95% CI (percent)	p
**Predicted risk of death due to dementia**			
Effect of vaccine receipt	2008	−30.6 (−67.2, 6.1)	0.102
	1706	−48.8 (−79.7, −17.9)	0.002
Effect of vaccination eligibility	2008	−12.9 (−32.0, 6.1)	0.184
	1706	−16.7 (−33.0, −0.5)	0.044
**Predicted risk of all−cause mortality**			
Effect of vaccine receipt	2306	−33.3 (−66.3, −0.3)	0.048
	1587	−49.1 (−82.8, −15.4)	0.004
Effect of vaccination eligibility	2306	−12.2 (−27.8, 3.4)	0.126
	1587	−18.5 (−37.2, 0.2)	0.053
**Length of dementia−related hospital stay**			
Effect of vaccine receipt	2131	−18.4 (−62.9, 26.1)	0.418
	1615	−58.7 (−84.6, −32.8)	0.000
Effect of vaccination eligibility	2131	−6.9 (−25.7, 12.0)	0.475
	1615	−22.3 (−38.7, −5.8)	0.008
**Number of dementia−related hospital admissions**			
Effect of vaccine receipt	2665	−35.2 (−63.3, −7.1)	0.014
	1073	−49.6 (−107.7, 8.4)	0.094
Effect of vaccination eligibility	2665	−14.6 (−29.6, 0.4)	0.057
	1073	−11.6 (−34.2, 11.0)	0.314
**Number of recordings of dementia**			
Effect of vaccine receipt	2292	−35.8 (−68.8, −2.8)	0.033
	1478	−41.7 (−79.0, −4.5)	0.028
Effect of vaccination eligibility	2292	−14.6 (−32.1, 2.9)	0.102
	1478	−13.6 (−30.8, 3.6)	0.122

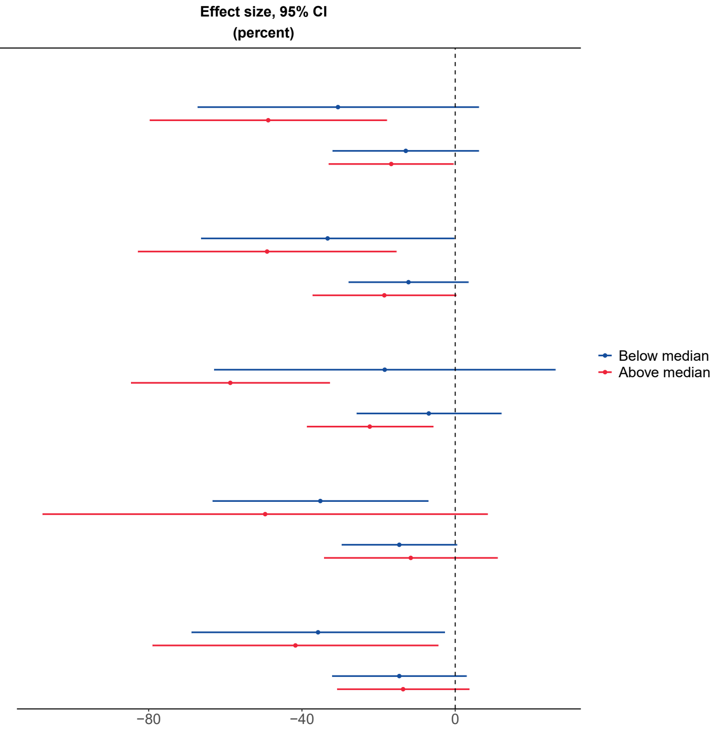

Figure S6. The absolute and relative effect of HZ vaccination eligibility and receipt on deaths due to dementia among those above versus below the median for different surrogate measures of dementia severity using regression discontinuity, related to Figure 4

Dots show the point estimate and horizontal bars the 95% CI. The definition and calculation of each dementia severity measure is provided in the STAR Methods. Each dementia severity measure was assessed during the time period prior to September 1, 2013. *p* values and CIs for the relative effect estimates were calculated using the delta method. There is, however, no well-established method for estimating *p* values and CIs for relative effect estimates in a regression discontinuity approach.[42] These values should, therefore, be interpreted with caution.

Abbreviation is as follows: CI, confidence interval.

Dementia severity measure	N	Effect size, 95% CI (percentage points)	p
**Predicted risk of death due to dementia**			
Effect of vaccine receipt	4334<br>4215	−33.5 (−68.7, 1.6)<br>−36.1 (−80.6, 8.3)	0.061<br>0.111
Effect of vaccination eligibility	4334<br>4215	−9.6 (−19.4, 0.3)<br>−8.3 (−18.4, 1.9)	0.056<br>0.112
**Predicted risk of all−cause mortality**			
Effect of vaccine receipt	4183<br>4366	−13.7 (−54.6, 27.2)<br>−53.1 (−91.6, −14.7)	0.511<br>0.007
Effect of vaccination eligibility	4183<br>4366	−3.3 (−13.2, 6.6)<br>−14.5 (−24.7, −4.4)	0.511<br>0.005
**Length of dementia−related hospital stay**			
Effect of vaccine receipt	4140<br>4409	−13.4 (−43.6, 16.7)<br>−75.7 (−137.6, −13.7)	0.383<br>0.017
Effect of vaccination eligibility	4140<br>4409	−4.3 (−14.1, 5.4)<br>−13.5 (−23.8, −3.2)	0.385<br>0.010
**Number of dementia−related hospital admissions**			
Effect of vaccine receipt	5927<br>2622	−25.7 (−53.3, 1.8)<br>−94.8 (−227.6, 38.1)	0.067<br>0.162
Effect of vaccination eligibility	5927<br>2622	−7.8 (−16.0, 0.5)<br>−10.7 (−24.5, 3.2)	0.066<br>0.131
**Number of recordings of dementia**			
Effect of vaccine receipt	5045<br>3504	−26.7 (−60.1, 6.8)<br>−54.4 (−103.7, −5.0)	0.119<br>0.031
Effect of vaccination eligibility	5045<br>3504	−7.2 (−16.2, 1.8)<br>−12.8 (−24.1, −1.5)	0.117<br>0.026

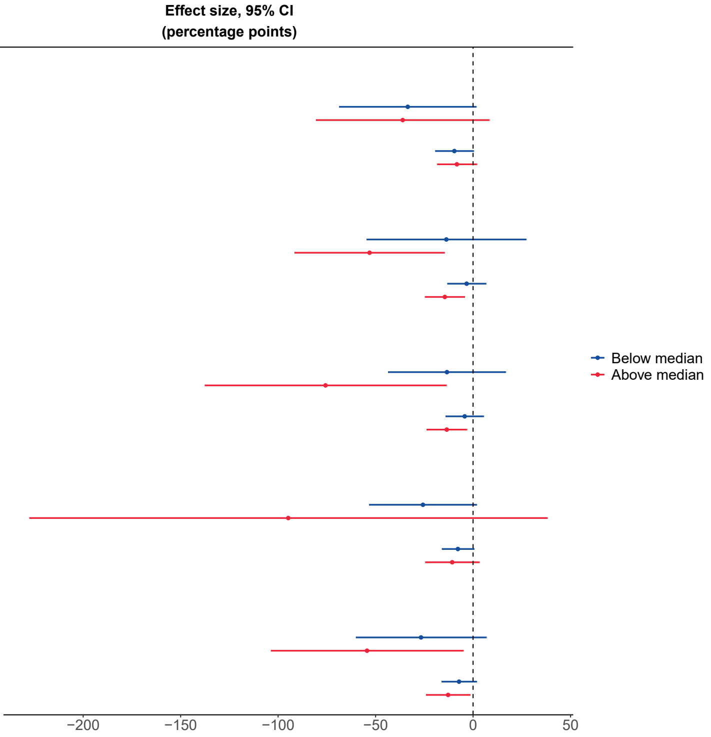

Figure S7. The absolute effect of HZ vaccination eligibility and receipt on deaths due to dementia among those above versus below the median for different surrogate measures of dementia severity using a difference-in-differences approach, related to Figure 4

Dots show the point estimate and horizontal bars the 95% CI. The definition and calculation of each dementia severity measure is provided in the STAR Methods. Each dementia severity measure was assessed during the time period prior to September 1, 2013.

Abbreviation is as follows: CI, confidence interval.

Women

	Effect size, 95% CI (percentage points)	p
**Deaths due to dementia**		
Effect of vaccine receipt	−52.3 (−97.9, −9.2)<br>−62.1 (−101.5, −22.7)	0.018<br>0.002
Effect of vaccination eligibility	−13.9 (−26.3, −3.4)<br>−14.9 (−23.9, −5.8)	0.011<br>0.001
**Non−dementia deaths**		
Effect of vaccine receipt	12.5 (−20.6, 45.4)<br>16.7 (−14.6, 47.9)	0.462<br>0.296
Effect of vaccination eligibility	3.3 (−5.1, 12.0)<br>4.0 (−3.4, 11.3)	0.434<br>0.288
**All−cause mortality**		
Effect of vaccine receipt	−34.7 (−63.0, −9.8)<br>−28.9 (−53.8, −3.9)	0.007<br>0.023
Effect of vaccination eligibility	−9.1 (−16.7, −3.1)<br>−6.9 (−12.8, −1.0)	0.004<br>0.023

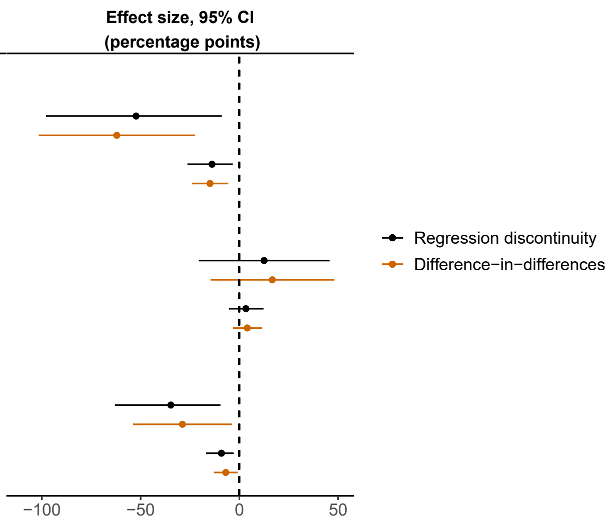

Men

	Effect size, 95% CI (percentage points)	p
**Deaths due to dementia**		
Effect of vaccine receipt	4.3 (−40.1, 41.6)<br>1.4 (−38.9, 41.7)	0.970<br>0.944
Effect of vaccination eligibility	1.3 (−12.4, 13.0)<br>0.4 (−11.0, 11.8)	0.966<br>0.944
**Non−dementia deaths**		
Effect of vaccine receipt	−9.2 (−41.4, 24.0)<br>−13.4 (−49.2, 22.4)	0.602<br>0.465
Effect of vaccination eligibility	−2.7 (−12.0, 7.1)<br>−3.8 (−13.9, 6.3)	0.614<br>0.462
**All−cause mortality**		
Effect of vaccine receipt	−4.1 (−29.7, 17.5)<br>−8.3 (−30.0, 13.3)	0.613<br>0.450
Effect of vaccination eligibility	−1.3 (−9.4, 5.5)<br>−2.4 (−8.6, 3.8)	0.609<br>0.453

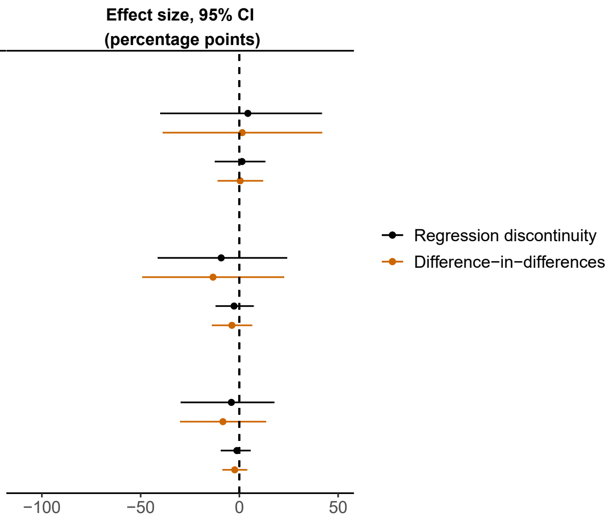

Figure S8. The effect of HZ vaccination eligibility and receipt on deaths due to dementia, non-dementia deaths, and all-cause mortality separately among women and men, related to Figure 5

Dots show the point estimate and horizontal bars the 95% CI. All outcomes were analyzed among a study cohort of patients who had received a diagnosis of dementia prior to the start date of the HZ vaccination program. Non-dementia deaths were defined as deaths for which dementia was neither the underlying nor a contributing cause of death in the death certificate.

Abbreviation is as follows: CI, confidence interval.

CellPress
OPEN ACCESS

Effect on all−cause mortality

	Effect size, 95% CI (percentage points)	p
**Bandwidth choice (as x−times the MSE−optimal bandwidth)**		
2	−4.3 (−8.6, −1.2)	0.010
	−16.4 (−33.3, −4.9)	0.008
1.75	−4.4 (−9.1, −1.2)	0.010
	−16.8 (−35.2, −5.1)	0.009
1.5	−4.6 (−9.7, −1.1)	0.014
	−17.4 (−36.4, −4.4)	0.013
1.25	−5.3 (−10.6, −1.2)	0.014
	−19.2 (−37.7, −4.0)	0.015
1	−6.5 (−12.5, −2.1)	0.006
	−22.7 (−42.8, −6.5)	0.008
0.75	−8.1 (−15.5, −3.4)	0.002
	−28.5 (−53.8, −11.1)	0.003
0.5	−9.1 (−17.5, −2.6)	0.008
	−32.9 (−63.5, −9.6)	0.008
**Grace−period (months)**		
4	−8.4 (−15.7, −3.1)	0.003
	−29.1 (−54.1, −9.2)	0.006
2	−7.2 (−13.6, −2.4)	0.005
	−24.9 (−46.6, −7.3)	0.007
0	−6.5 (−12.5, −2.1)	0.006
	−22.7 (−42.8, −6.5)	0.008
**Other checks**		
Quadratic fit	−7.7 (−14.5, −2.5)	0.006
	−25.9 (−47.5, −7.3)	0.008
Adjusting for baseline IHD diagnosis	−3.2 (−6.0, −0.5)	0.022
	−12.3 (−22.7, −1.9)	0.020
Accounting for staggered roll−out	−6.8 (−13.1, −2.3)	0.005
	−26.1 (−50.3, −8.3)	0.006

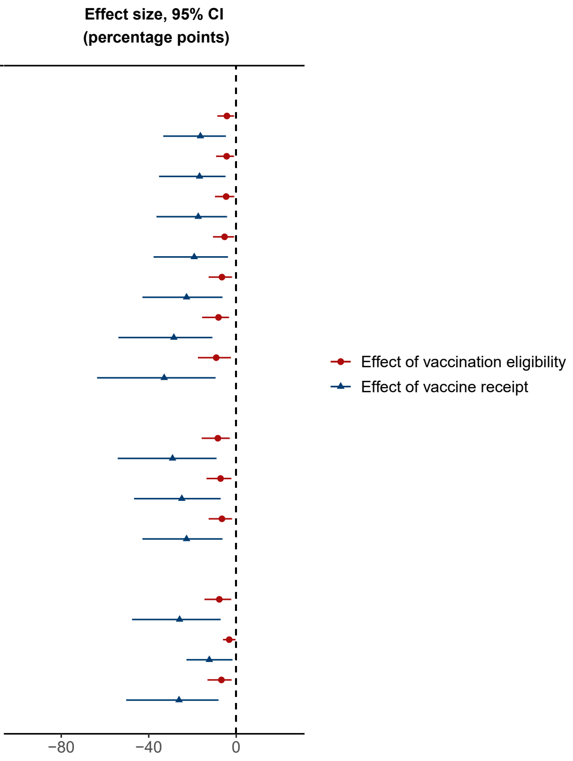

Effect on all−cause mortality among women

	Effect size, 95% CI (percentage points)	p
**Bandwidth choice (as x−times the MSE−optimal bandwidth)**		
2	−6.9 (−12.7, −2.7)	0.002
	−29.0 (−53.4, −11.4)	0.003
1.75	−7.4 (−13.8, −3.3)	0.002
	−30.8 (−58.0, −13.5)	0.002
1.5	−7.5 (−14.5, −3.1)	0.002
	−31.0 (−59.3, −12.3)	0.003
1.25	−7.9 (−15.0, −2.7)	0.005
	−31.5 (−59.3, −9.4)	0.007
1	−9.1 (−16.7, −3.1)	0.004
	−34.7 (−63.0, −9.8)	0.007
0.75	−10.9 (−20.2, −4.8)	0.002
	−40.9 (−75.6, −15.2)	0.003
0.5	−12.8 (−23.8, −5.2)	0.002
	−48.4 (−92.2, −17.2)	0.004
**Grace−period (months)**		
4	−10.9 (−19.8, −4.2)	0.002
	−41.2 (−75.3, −12.7)	0.006
2	−11.8 (−20.3, −5.5)	0.001
	−44.4 (−77.2, −17.3)	0.002
0	−9.1 (−16.7, −3.1)	0.004
	−34.7 (−63.0, −9.8)	0.007
**Other checks**		
Quadratic fit	−9.2 (−16.8, −2.3)	0.010
	−34.8 (−63.1, −6.8)	0.015
Adjusting for baseline IHD diagnosis	−8.1 (−12.1, −4.0)	<0.001
	−33.4 (−50.2, −16.6)	<0.001
Accounting for staggered roll−out	−8.5 (−16.4, −2.3)	0.010
	−34.8 (−66.7, −7.6)	0.014

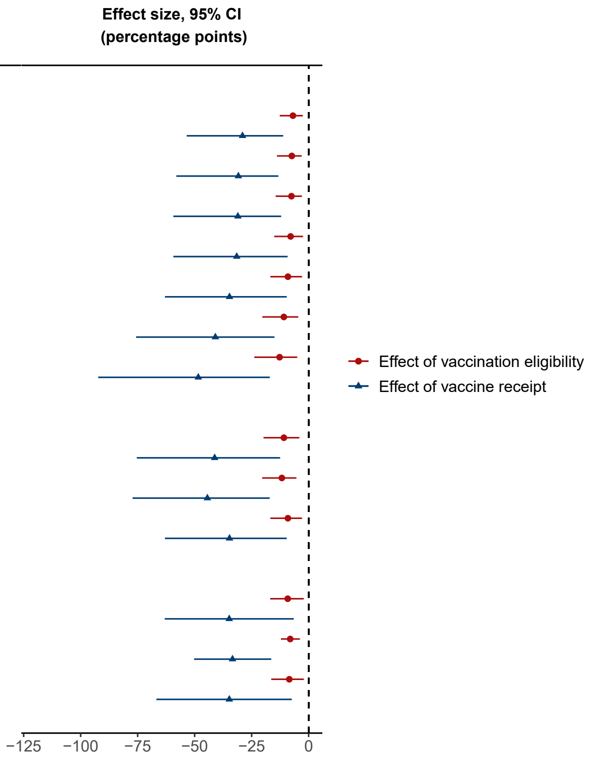

(legend on next page)

Figure S9. Robustness checks for the effect of HZ vaccination eligibility and receipt on all-cause mortality among patients living with dementia at baseline, related to Figure 5

Baseline refers to the start date of the HZ vaccination program (i.e., September 1, 2013). Dots or triangles show the point estimate and horizontal bars the 95% CI. With grace periods, we refer to time periods since the index date after which follow-up time is considered to begin to allow for the time needed for a full immune response to develop after vaccine administration. As described in detail in the STAR Methods section, when accounting for the staggered roll-out of the program, we adjusted the follow-up period to begin for each individual on the date on which they first became eligible for HZ vaccination (instead of starting the follow-up period for all individuals on September 1, 2013). We added cohort fixed effects to these analyses to control for between-cohort differences in the date at which the follow-up window started. That is, we defined one cohort fixed effect for ineligible individuals and the first catch-up cohort and included additional cohort fixed effects for each group of patients who became eligible at the same time.

Abbreviations are as follows: CI, confidence interval; MSE, mean squared error; IHD, ischemic heart disease.

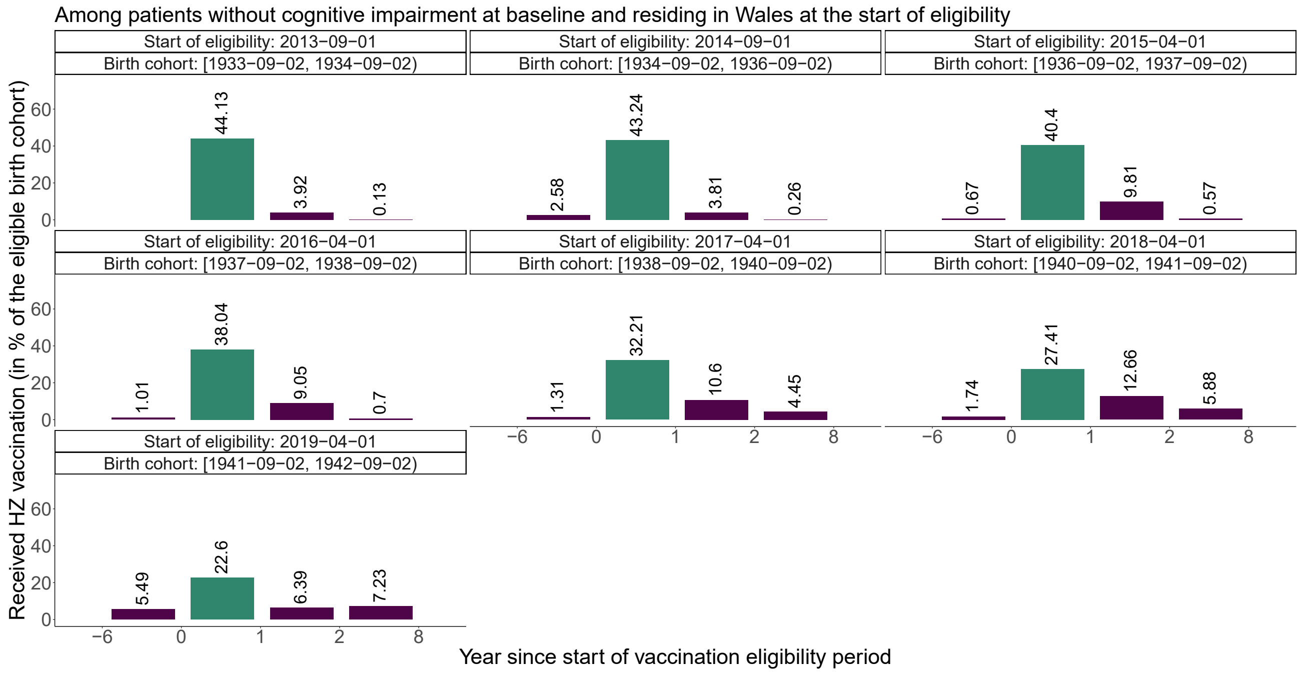

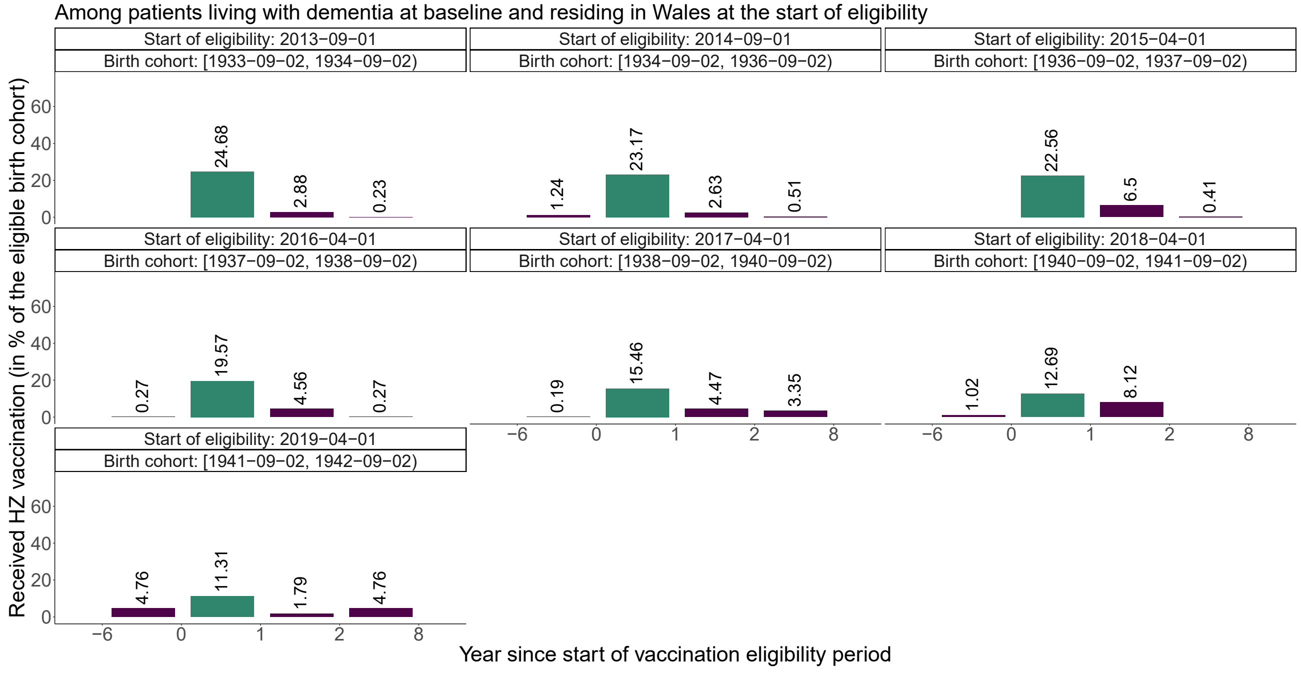

Figure S10. Uptake of herpes zoster vaccination by birth cohort and year of eligibility, related to Figure 1

Baseline refers to the start date of the HZ vaccination program (i.e., September 1, 2013). The green bar corresponds to the respective first year of eligibility for a particular cohort. Eligibility years 2 to 8 had to be aggregated to comply with SAIL data publication standards.

Abbreviation is as follows: HZ, herpes zoster.

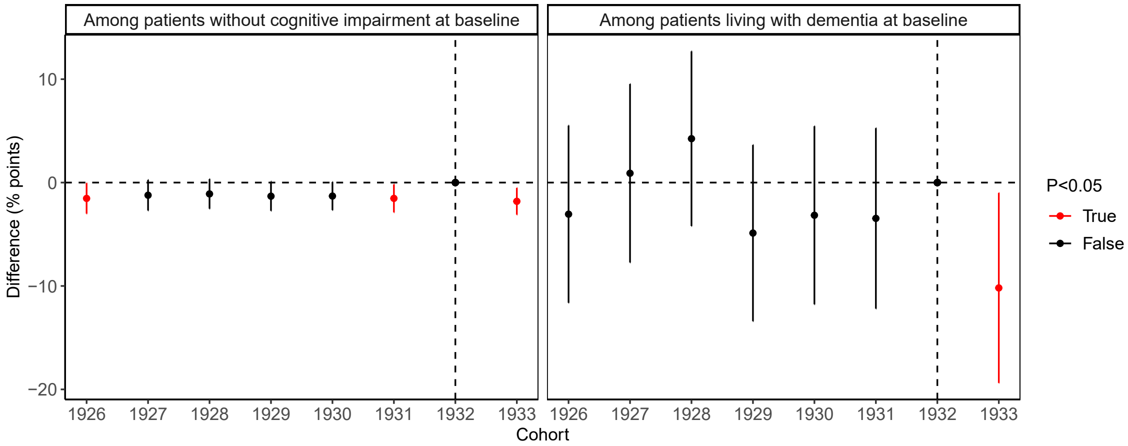

Figure S11. Difference in the incidence of our primary outcomes between the pre- and post-September birth "seasons" for each yearly birth cohort, related to STAR Methods section "Evidence triangulation"

Baseline refers to the start date of the HZ vaccination program (i.e., September 1, 2013). "Difference" refers to the difference between the pre- and post-September birth seasons. The pre-September birth season was defined as the 6-month period from March 1 to August 31 of a given year and the post-September birth season as the 6-month period from September 1 to February 28 of the succeeding year. The outcome was new diagnoses of MCI for the study cohort among patients without cognitive impairment at baseline, and deaths due to dementia for the study cohort among patients living with dementia at baseline.

The 1932/1933-cohort served as the reference cohort. Statistically significant differences (indicated in red) for birth-year cohorts other than the 1933/1934 cohort indicate that the assumption needed for an unbiased difference-in-differences analysis may not be met. The regression equations for this analysis are detailed in the STAR Methods.
